# From mutation detection to treatment decisions: CRISPR-enabled diagnostics for drug-resistant tuberculosis

**DOI:** 10.3389/fbioe.2026.1947704

**Published:** 2026-09-17

**Authors:** Weijia Yi, Fukang Luo, Dianping Yi, Jing Guo, Shan Liu, Qibing Yan, Hongzhi Luo

**Affiliations:** 1 West China Hospital Sichuan University Jintang Hospital, Jintang First People’s Hospital, Chengdu, China; 2 The Fourth People’s Hospital of Jintang (Jintang County Mental Health Center), Chengdu, China; 3 Genetic Diseases Key Laboratory of Sichuan Province, Department of Medical Genetics, Sichuan Academy of Medical Sciences and Sichuan Provincial People’s Hospital, University of Electronic Science and Technology of China, Chengdu, China

**Keywords:** antimicrobial resistance, clinical translation, CRISPR diagnostics, drug-resistant tuberculosis, heteroresistance, mutation-to-drug interpretation

## Abstract

Effective management of drug-resistant tuberculosis requires molecular assays that not only detect *Mycobacterium tuberculosis* but also identify resistance-associated variants, support drug-level interpretation, and indicate when broader confirmatory testing is needed. This narrative review applies a mutation-to-drug-to-decision framework to distinguish assay validity, exact-variant detection, drug-level inference, and decision impact. We synthesize evidence on target accessibility, minority-allele preservation, multiplexing, direct-specimen testing, and sequencing enrichment, emphasizing how these factors shape clinical interpretability. Current evidence remains dominated by analytical studies, isolate-based evaluations, and small direct-specimen investigations, with prospective multicenter treatment-impact validation still lacking. Population-level benchmarks indicate incomplete biological coverage for compact target regions, reaching approximately 92.6% for *rpoB* RRDR-based rifampicin detection, 85.4% for *katG* codon 315 plus the *inhA* promoter for isoniazid, and 84.7%–88.2% for *gyrA/gyrB*-based fluoroquinolone detection. Allele-specific CRISPR is therefore best suited to rapid rule-in of selected resistance-associated variants, while dispersed or heterogeneous mechanisms require broader molecular or phenotypic assessment. CRISPR should complement WHO-recommended rapid molecular testing and targeted next-generation sequencing rather than replace established molecular or phenotypic methods.

## 1 Introduction

Tuberculosis remains a leading infectious cause of morbidity and mortality worldwide (Burki, 2024; Farhat et al., 2024; World Health Organization, 2025a), and drug-resistant tuberculosis continues to compromise treatment success, transmission control, and public health resource allocation (Farhat et al., 2024; Dheda et al., 2017). The growing use of shorter all-oral regimens has increased the clinical importance of rapid resistance information for regimen-defining drugs, including rifampicin, isoniazid, fluoroquinolones, bedaquiline, linezolid, delamanid, and pretomanid (World Health Organization, 2025b; Günther et al., 2024; Nyang’wa et al., 2022; Conradie et al., 2022). Under current definitions, pre-extensively drug-resistant tuberculosis requires multidrug- or rifampicin-resistant tuberculosis with additional fluoroquinolone resistance, whereas extensively drug-resistant tuberculosis also requires resistance to at least one of bedaquiline and linezolid (Viney et al., 2021). Diagnostic panels must therefore evolve with treatment recommendations and resistance epidemiology rather than remain restricted to historical first-line targets.

Molecular diagnosis of tuberculosis involves two related but distinct tasks. Pathogen confirmation establishes the presence of the *Mycobacterium tuberculosis* complex and informs treatment initiation in the appropriate clinical context (Boehme et al., 2010; Dorman et al., 2018). Resistance interpretation asks a different question: which drugs are likely to be ineffective, which regimens should be avoided, and which additional tests are required (Nahid et al., 2019; Liebenberg et al., 2022; Heyckendorf et al., 2018; Yusoof et al., 2022). A method optimized only for a conserved pathogen target may perform well for tuberculosis detection but remain unsuitable for resistance testing, in which single-nucleotide discrimination, target coverage, and preservation of minority-variant signals or allele fractions determine clinical value.

Phenotypic drug susceptibility testing remains an important reference because it measures growth under drug exposure, but it is culture dependent and requires time, biosafety infrastructure, and rigorous quality assurance (Heyckendorf et al., 2018; Yusoof et al., 2022). Current WHO diagnostic guidance places rapid molecular testing at the center of initial tuberculosis and rifampicin-resistance detection and includes follow-on molecular drug-susceptibility testing, including targeted next-generation sequencing (tNGS), for broader resistance characterization (World Health Organization, 2025c). Xpert MTB/RIF and Xpert Ultra provide rapid tuberculosis and rifampicin-resistance detection, while Xpert MTB/XDR extends coverage to selected loci associated with isoniazid, fluoroquinolone, ethionamide, and injectable-drug resistance (Pfurtscheller et al., 2025; Penn-Nicholson et al., 2022; Horne et al., 2025; Pillay et al., 2022; Cao et al., 2021). Line-probe assays detect common resistance loci, whereas tNGS and whole-genome sequencing provide progressively broader, updateable mutation profiles (Nathavitharana et al., 2017; Schwab et al., 2024; Colman et al., 2025; Walker et al., 2015). These methods have transformed care, but fixed target content, variable bacillary burden, cost, workflow complexity, bioinformatic requirements, and interpretation of rare variants remain important constraints (Walzl et al., 2018; Naidoo and Dookie, 2022; Nguyen et al., 2019; Zifodya et al., 2021; Kohli et al., 2021; Detjen et al., 2015).

Despite these advances, there remains a need for flexible assay platforms that can identify clinically actionable resistance variants, preserve low-frequency mutant signals in direct specimens, and indicate the residual risk after a negative hotspot result. This need is particularly evident in smear-negative or paucibacillary disease, mixed bacterial populations, treatment failure, and regions where fixed commercial panels do not adequately represent local or emerging resistance mechanisms.

CRISPR-associated systems offer programmable target recognition and can be combined with polymerase chain reaction, isothermal amplification, digital partitioning, lateral-flow readout, and electrochemical sensing (Gootenberg et al., 2017; Chen et al., 2018; Gootenberg et al., 2018; Kellner et al., 2019; Politza et al., 2023; Wulandari et al., 2025). Studies of tuberculosis detection have evaluated proof-of-concept detection across respiratory specimens, cerebrospinal fluid, pediatric stool, blood- or plasma-derived targets, and tongue swabs (Ai et al., 2019; Sam et al., 2021; Peng et al., 2024; Huang et al., 2025; Bell et al., 2025; Youngquist et al., 2025; Thakku et al., 2023; Huang Z. et al., 2022; Liu et al., 2025a; Yu et al., 2026). Recent assay-engineering work has also explored crRNA scaffold remodeling for programmable Cas12a control (Wang et al., 2026) and optothermal paperfluidic integration for tuberculosis detection (Thepbut et al., 2026). These studies provide proof-of-concept engineering evidence for sensitive pathogen detection, but they do not automatically establish mutation-level or drug-level validity for resistance testing.

Previous reviews have generally organized CRISPR-based tuberculosis diagnostics by effector type, amplification method, analytical sensitivity, or point-of-care format (Abavisani et al., 2024; Huang et al., 2023; Qi et al., 2022; Zein-Eddine et al., 2023). For resistance testing, the central question is the clinical meaning of a CRISPR signal. This narrative review therefore examines which assay designs preserve and discriminate resistance-associated alleles, which variants support drug-level interpretation, and whether and how the resulting information could alter confirmatory testing or treatment decisions. These questions are considered within a mutation-to-drug-to-decision framework that links molecular engineering, target selection, direct-specimen evidence, heteroresistance, multiplexing, reporting, and implementation. The overall progression from heterogeneous clinical specimens through programmable CRISPR detection to staged clinical positioning is summarized in Figure 1.

**FIGURE 1:**
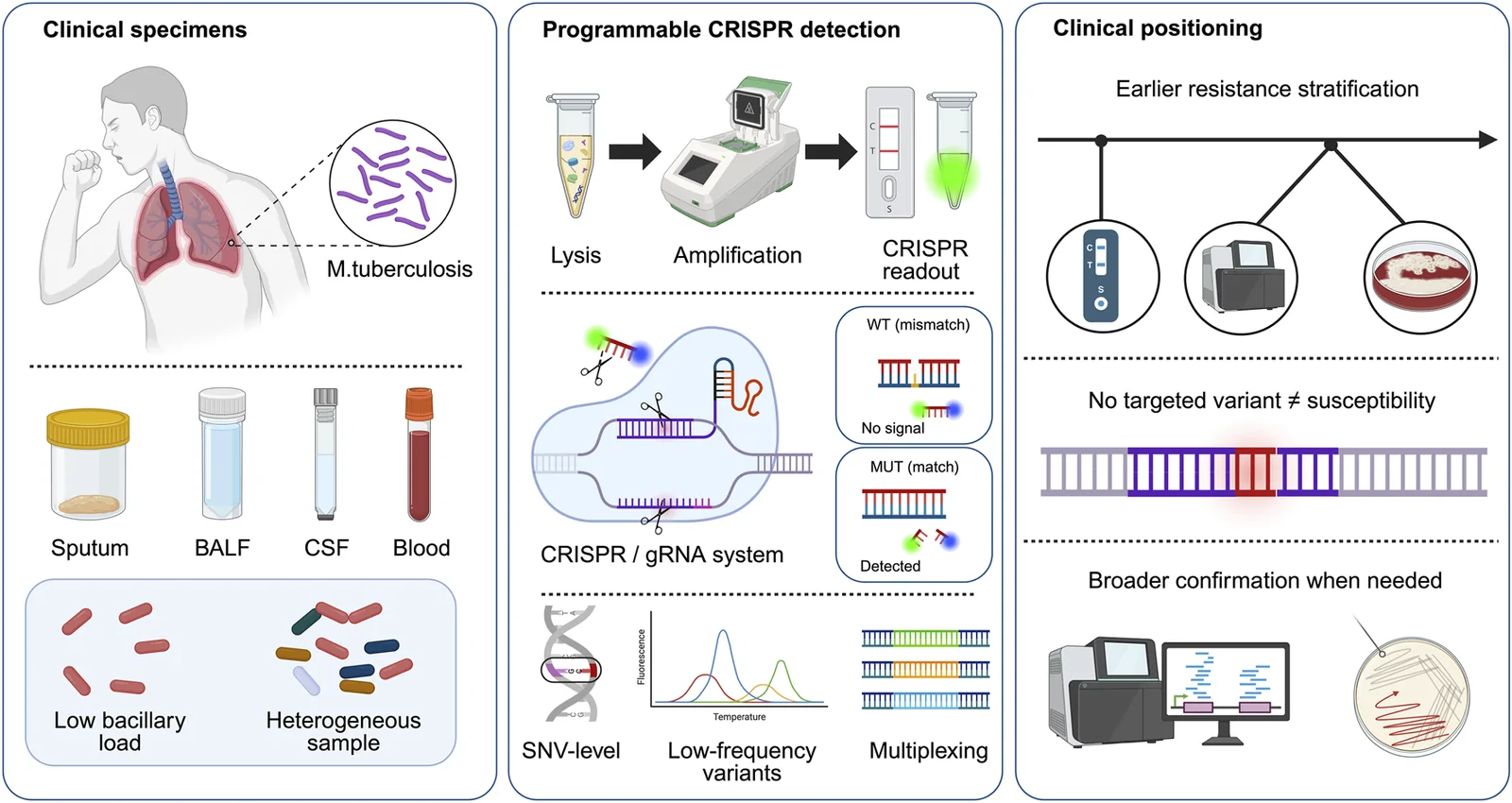
Author-generated conceptual overview of CRISPR-enabled resistance testing for tuberculosis. This conceptual framework is not a validated consensus diagnostic algorithm. Abbreviations: BALF, bronchoalveolar lavage fluid; CSF, cerebrospinal fluid; gRNA, guide RNA; MUT, mutant; SNV, single-nucleotide variant; WT, wild type.

### 1.1 Literature identification and evidence selection

This narrative review was informed by a structured literature search from database inception through 24 July 2026. PubMed was the primary bibliographic database, supplemented by Google Scholar, publisher-platform searches, and backward/forward citation tracking. Search terms combined tuberculosis/*Mycobacterium tuberculosis*, CRISPR/Cas, drug resistance/resistance variants, heteroresistance, multiplexing, direct specimens, and sequencing enrichment. We included peer-reviewed original studies relevant to CRISPR-enabled tuberculosis detection or resistance testing; preprints and conference abstracts were excluded, and reviews were used only for contextual background. No language restriction was applied during study identification. Resistance-specific studies were distinguished from broader pathogen-detection or engineering studies. Table 1 prioritizes representative studies by specimen type, reference standard, and highest supported diagnostic claim. No pooled meta-analysis was performed.

**TABLE 1:** Representative core studies relevant to mutation-resolved drug-resistant tuberculosis testing.

Study/approach	Targets	Design/specimens	Reference standard	Performance (95% CI)	Workflow/analytical note	Highest supported claim and principal limitation
Engineered Cas12a RR - *rpsL* (Liu et al., 2022a)	*rpsL* p.Lys43Arg/p.Lys88Arg	Constructed targets and simulated mixtures	Designed sequence/mixture composition	Minority fraction ∼0.1%; no clinical accuracy CI	Analytical only	Low-frequency variant discrimination; no direct-specimen or clinical-action threshold
Engineered Cas12a RR - *katG* (Liu et al., 2025b)	*katG* p.Ser315Thr	42 cultured isolates: 27 c.944G>C-positive, 15 negative; controlled mixtures	Sequence-defined mutation status	27/27 sensitivity 100% (87.2–100); 15/15 specificity 100% (78.2–100)	∼1% minority fraction after WT depletion; ∼60-min post-extraction workflow	Isolate-level exact-variant discrimination; one INH variant; WT depletion alters allele fractions
Asymmetric RPA-Cas12a (Cao et al., 2023)	Selected *rpoB* RIF variants	Simulated mixtures; model samples	Defined templates	∼1% mutant fraction; no clinical accuracy CI	PAM-independent ssDNA strategy; LoD 1 fM; 2 µL input ≈1.2 × 10^3 target molecules/reaction	Analytical discrimination; no direct-specimen validation
Multiplex RPA-AaCas12b (Hu et al., 2026)	IS6110; *rpoB* c.1349C>T; *katG* c.944G>C	48 residual pretreated sputum/BALF specimens	Sequencing for exact variants; pDST for drug phenotype	*rpoB*: Se 94.1% (16/17; 71.3–99.9), Sp 100% (31/31; 88.8–100). *katG*: Se 94.7% (18/19; 74.0–99.9), Sp 93.1% (27/29; 77.2–99.2). Phenotype Se lower	∼40-min preparation +30–35-min assay/readout	Direct-specimen exact-mutation detection; one variant/drug and readout-threshold dependence limit phenotype inference
Cas13a *gyrA* assay (Bai et al., 2022)	Seven common *gyrA* variants	75 cultured clinical isolates (35 FQ-resistant; 40 susceptible)	pDST	Se 91.4% (32/35; 76.9–98.2); Sp 100% (40/40; 91.2–100)	Isolate DNA; no direct sputum	Targeted FQ-resistance rule-in; incomplete *gyrB*/mechanism coverage
Multiplex PCR-Cas14a - Xiao (Xiao et al., 2025)	*rpoB*, *katG*, *inhA* hotspots	60 isolates; 55 smear-positive, culture-positive sputum specimens	pDST and sequence interpretation	Sputum RIF: Se 83.3% (10/12; 51.6–97.9), Sp 100% (43/43; 91.8–100). INH: Se 100% (11/11; 71.5–100), Sp 100% (44/44; 92.0–100)	∼2.5 h including extraction; post-PCR open transfer	Direct-sputum hotspot detection in high-bacillary-load specimens; source reports 85.7% for 10/12; isolate arm reports 93.3% for 39/42 (arithmetic 92.9%); D435Y miss despite nominal guide inclusion; L452P not covered
Asymmetric PCR-Cas14a - Wu (Wu et al., 2025a)	Eight prevalent *rpoB* RRDR mutation types + MTBC target	16 cultured clinical isolates; 128 blinded reactions	Sequencing	Complete reaction-level concordance reported; phenotype-class CI not derivable	Extracted isolate DNA; separate Cas reactions	Multigenotype isolate concordance; no direct-specimen or independent drug-phenotype evaluation
CARP (Augustin and Agarwal, 2023)	*rpoB* p.Ser450Trp (legacy S531W) and p.His445Cys (legacy H526C)	Designed WT/mutant amplicons; no clinical cohort	Defined templates/sequencing-confirmed constructs	Analytical discrimination only	∼2 h Cas9 cleavage + ∼2 h qPCR; source 0.0001 fg genomic DNA ≈2 × 10^-5 H37Rv genome equivalents (below plausible sampling; not treated as a physical LoD)	Proof-of-concept variant discrimination; two-variant coverage and no clinical validation
ACURAT (Zheng et al., 2024)	15 *rpoB* genotypes at modern codons 435/445/450/452 (legacy 516/526/531/533)	60 culture-derived clinical isolates collected (10 drug-sensitive; 50 RIF/INH-resistant); CRISPR cross-validation used selected WT + 6 mutant isolates	MIC/pDST for resistance; Sanger sequencing for genotype	No full-cohort diagnostic accuracy estimate for ACURAT; 50 resistant isolates used primarily for local RRDR-genotype mapping	Nested PCR + PAM-PCR + Cas9; ∼3-h workflow described	Expanded *rpoB* genotyping and regional panel design; isolate/culture-derived evidence, not direct-specimen diagnostic accuracy
Multi-AS-PCR-Cas12a (Wu et al., 2024)	Ten *rpoB* variants	24 sputum/BALF specimens: 9 RIF-resistant, 15 RIF-sensitive	Sanger sequencing	Se 100% (9/9; 66.4–100); Sp 93.3% (14/15; 68.1–99.8)	Nested PCR + Cas12a; LoD 10^2 aM (∼10^2 plasmid copies/µL); 5 µL input ≈3 × 10^2 molecules/reaction; total sample-to-result time not reported	Expanded targeted respiratory-specimen detection; ten primers required C4/C6 grouping because of competition; one false positive
FLASH-TB (Tram et al., 2023)	Identification regions +52 candidate resistance genes	16 direct sputum specimens	Sequencing	Resistance prediction possible in 15/16 (93.8%; 69.8–99.8), not a conventional sensitivity estimate	CRISPR-Cas9 sequencing enrichment	Improves direct-sputum sequencing recovery; sequencing dependent, small cohort, enrichment-uniformity requirements
RCA-Cas12a-Multi-SNV cassette (Wu et al., 2025b)	Four *rpoB* resistance-associated SNVs	16 sputum/BALF specimens	Sanger sequencing	Source reports Se 100%, Sp 83.3%, PPV 90.9%, NPV 100%. If counts implied by percentages are used: Se 10/10 (69.2–100), Sp 5/6 (35.9–99.6); displayed table labels/totals are internally inconsistent	One-tube ligation/RCA/Cas12a; LoD 10^4 aM (10 fM); 20-µL reaction ≈1.2 × 10^5^ target copies/reaction; extraction not integrated; source timing varies	One-tube targeted RIF screen; four-variant coverage, source-data inconsistency, limited clinical set
Thin-film microfluidic CRISPR (Cheng et al., 2026)	Seven *rpoB* RRDR variants	28 clinical specimens; specimen composition not clearly reported	Sanger sequencing	Full concordance reported; class-specific counts and 95% CI not derivable	18-min extraction +45-min AS-PCR +15-min CRISPR = 78 min; 1 CFU/mL on-chip. At 0.1 copies/µL, 20 µL extract ≈2 total copies (∼0.4 mutant copy at 20%); strongly sampling-dependent	Integrated sample-to-result genotyping; restricted panel, unclear specimen composition, no phenotype-level sensitivity
Cas9n/Cas9 self-priming assay - Gan (Gan et al., 2026)	*rpoB* RRDR	20 cultured clinical isolates: 6 RIF-resistant, 14 susceptible	Phenotypic DST + PCR/Sanger confirmation	Se 100% (6/6; 54.1–100); Sp 100% (14/14; 76.8–100)	LoD 0.74 fM; 20 µL reaction ≈8.9 × 10^3 target molecules/reaction; 100-min self-priming amplification + additional steps	Isolate-level RIF screening; resistant mutation spectrum not enumerated; no direct specimens
RPA-Cas12a *rpoB* p.Leu378Arg (Yang et al., 2026)	*rpoB* p.Leu378Arg	Constructed/reference genomic materials	Defined genotype	Variant-specific analytical performance only	Single-variant analytical assay	Technical programmability only; WHO Group 3 uncertain significance (World Health Organizatio n, 2023) and no clinical drug-phenotype validation
Electrochemical Cas9 *katG* sensor (Wulandari et al., 2025)	*katG* codon 315	Designed DNA targets	Defined templates	Source LoD 7.55 a.m.; copies/reaction not derivable from report	Analytical biosensor	Alternative sensing format; no direct clinical validation

Abbreviations: BALF, bronchoalveolar lavage fluid; FQ, fluoroquinolone; INH, isoniazid; PAM, protospacer-adjacent motif; PCR, polymerase chain reaction; pDST, phenotypic drug susceptibility testing; RIF, rifampicin; RPA, recombinase polymerase amplification; RRDR, rifampicin resistance-determining region; SNV, single-nucleotide variant. Exact 95% confidence intervals were calculated by the Clopper-Pearson method when explicit numerators and denominators were available; source-reported values were retained otherwise. Performance against sequencing refers to exact-variant concordance and should not be interpreted as drug-level sensitivity. Source analytical units are retained, and approximate molecules per reaction are added when both concentration and input volume are unambiguously reported. When conversion is not derivable, this is stated explicitly; source claims below plausible single-molecule sampling are flagged rather than treated as comparable physical LoDs. Cas14a nomenclature is retained where used in source publications; these systems are now generally classified within Cas12f.

## 2 From analytical signals to clinical claims

### 2.1 The mutation-to-drug-to-decision framework

Comparisons based only on the Cas effector, reaction time, or analytical limit of detection can be misleading. An assay may distinguish a prespecified mutant template with excellent analytical performance yet have low drug-level sensitivity if its panel covers only a limited proportion of resistant phenotypes. Conversely, broader variant coverage does not guarantee routine usability when the workflow involves open-tube transfer, amplification bias, inadequate internal controls, or uncertain reporting thresholds. The mutation-to-drug-to-decision framework keeps these claims separate and prevents evidence at one claim level from being extrapolated to the next (Table 2). It is an author-proposed interpretive model that complements, rather than replaces, WHO intended-use/product-evaluation approaches, target-product-profile concepts, mutation-catalogue interpretation, and STARD reporting principles (World Health Organization, 2025c; Bossuyt et al., 2015; World Health Organization, 2024). WHO frameworks primarily define intended use and expected diagnostic performance, whereas STARD addresses transparent reporting; the added purpose here is to distinguish assay validity, exact-variant detection, drug-level inference, and decision impact. In this review, levels refer to diagnostic claim domains, whereas stages refer to evidence maturity.

**TABLE 2:** Complementary dimensions for evaluating CRISPR-based tuberculosis diagnostics.

Dimension	Commonly emphasized platform dimension	Additional treatment-oriented consideration
Primary organizing principle	Cas effector, amplification method, or readout	Diagnostic claim and intended clinical use
Main analytical endpoint	Pathogen limit of detection or fluorescence signal	Variant discrimination and preservation of mutant allele fractions
Drug interpretation	Often secondary or implicit	Explicit mutation-to-drug inference with evidence tiers
Negative result	Presence or absence of the assay target	No targeted mutation within validated specimen and panel limits
Evidence categories	Evidence from templates, isolates, and specimens summarized by platform	Constructed, simulated, isolate, direct-specimen, and pathway evidence separated
Heteroresistance	Lowest detectable mutant fraction	Analytical, reproducible-reporting, and clinical-action thresholds considered separately
Clinical position	Rapid or point-of-care diagnostic potential	Rule-in triage, staged screening, or sequencing enrichment
Panel updating	Programmability as a general advantage	Surveillance-informed updates with analytical and clinical revalidation

Claim level 1, assay validity, evaluates whether the specimen contains an adequate amount of amplifiable *Mycobacterium tuberculosis* nucleic acid and whether extraction, amplification, and reaction controls have passed. Claim level 2, exact-variant detection, evaluates whether the assay accurately identifies a prespecified variant against an appropriate sequencing reference within the validated specimen and mutant-fraction range. Claim level 3, drug-level inference, evaluates whether the detected variant supports resistance to a drug when interpreted against genotype–phenotype evidence and, where technically validated and clinically interpretable, phenotypic drug susceptibility testing. Claim level 4, decision impact, evaluates whether use of the test changes treatment selection, referral, confirmatory testing, or time to an effective regimen.

Negative results also have claim-level-specific meaning. A valid negative mutation call indicates that no targeted variant was detected within the validated panel coverage. It does not establish phenotypic susceptibility when the bacillary input is low, the resistant subpopulation falls below the validated mutant-fraction threshold, amplification distorts allele proportions, regional variants are absent from the panel, or resistance mechanisms are dispersed across a gene. Reports therefore need to state these limits explicitly. The corresponding resistance rule-in, valid-negative-within-coverage, and unresolved-result pathways are shown in Figure 2.

**FIGURE 2:**
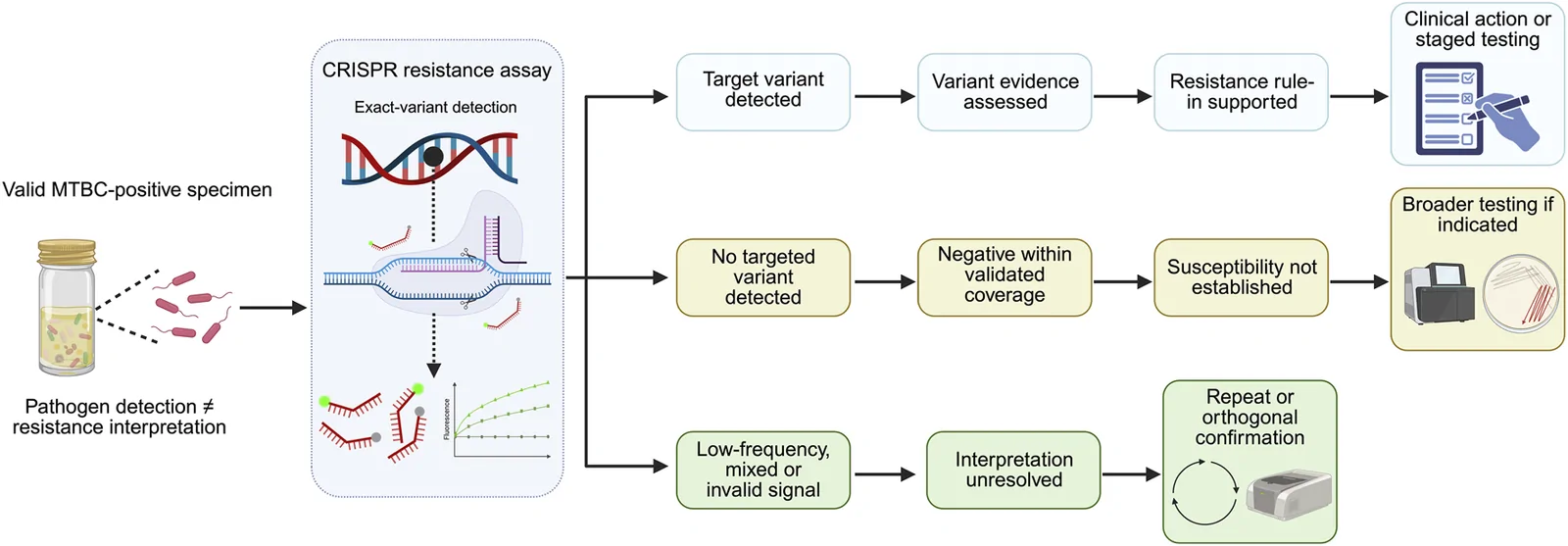
Author-generated conceptual interpretation pathways for an exact-variant CRISPR resistance assay in a valid *Mycobacterium tuberculosis* complex-positive specimen. This framework is not a consensus laboratory standard. Abbreviation: MTBC, *Mycobacterium tuberculosis* complex.

### 2.2 Evidence maturity and intended use

The evidentiary meaning of a CRISPR result depends strongly on the specimen and study design. Constructed oligonucleotides and plasmids are appropriate for guide screening and analytical discrimination. Simulated mixtures permit controlled assessment of mutant fractions and wild-type interference. Clinical isolates show whether target variants can be detected in naturally occurring genomes but bypass direct-specimen lysis, inhibition, and background DNA. Direct testing of sputum or other clinical specimens provides stronger evidence for diagnostic use, whereas prospective multicenter studies linked to treatment decisions are needed to establish pathway impact. These categories should be stated explicitly rather than pooled under the general label of clinical validation. Figure 3 summarizes evidence maturity as four stages: analytical evidence, clinical-isolate evidence, direct-specimen evidence, and clinical decision-impact evidence. For visual simplicity, Stage 1 combines molecular-engineering studies and simulated allele-mixture studies, which are separated as E1 and E2 in Supplementary Table S1. These evidence stages are distinct from the four claim levels defined in Section 2.1.

**FIGURE 3:**
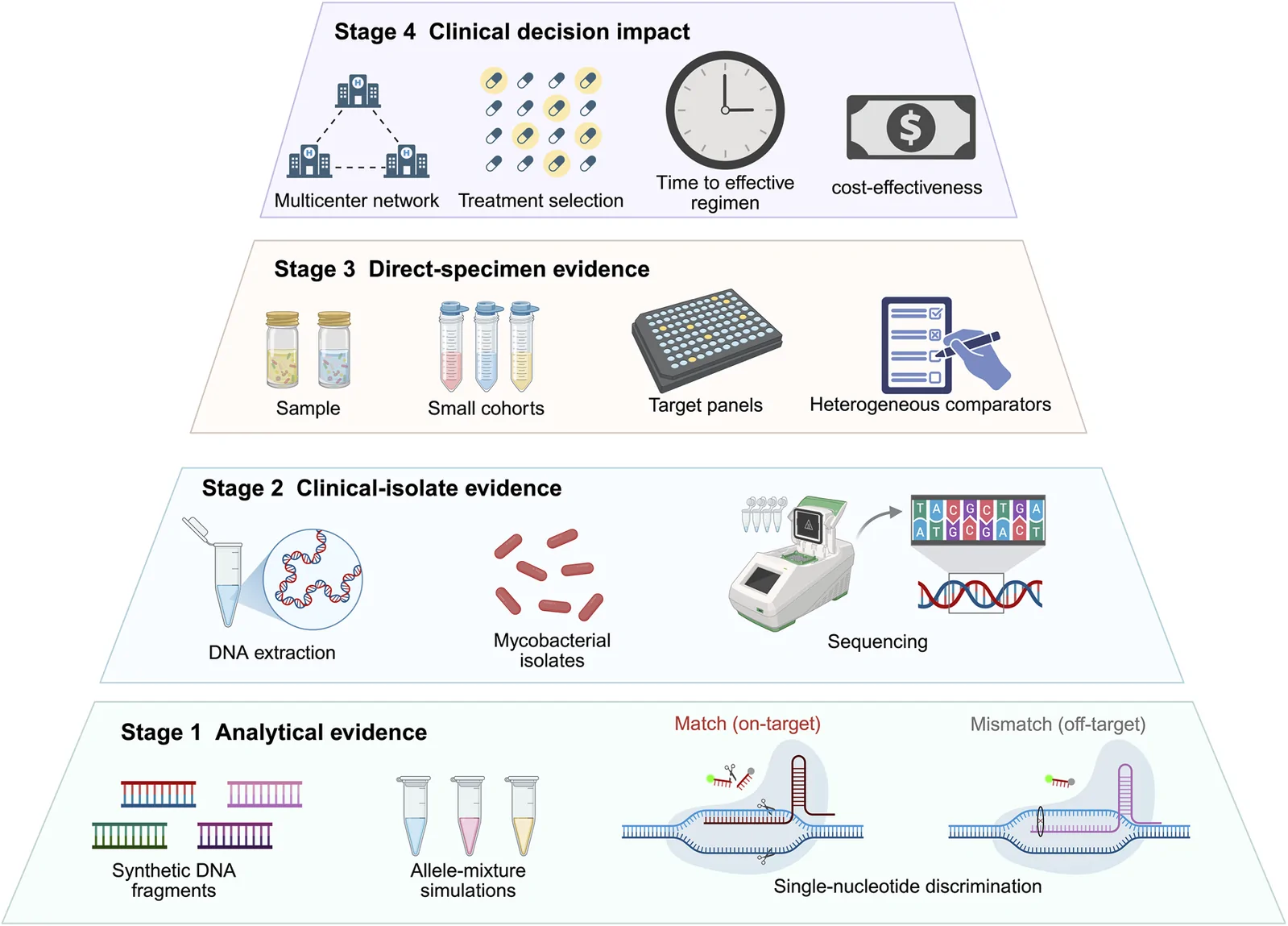
Author-generated conceptual four-stage evidence-maturity hierarchy for the clinical translation of CRISPR-enabled drug-resistant tuberculosis diagnostics.

At present, the evidence supports three main uses. Rapid rule-in panels can identify common, well-validated resistance-associated variants and trigger an expanded drug-resistant tuberculosis workup. Stepwise triage panels can screen regimen-defining targets before broader sequencing or phenotypic testing. CRISPR-guided enrichment can increase the proportion and depth of *Mycobacterium tuberculosis* reads in direct-specimen sequencing. Because these applications address different bottlenecks, they require different performance measures.

### 2.3 Target accessibility and single-nucleotide discrimination

A basic engineering challenge is reliable discrimination between wild-type and resistance-associated alleles at clinically relevant loci. Cas12a is widely used because of its simple guide architecture and collateral cleavage of single-stranded DNA reporters, but its canonical thymine-rich protospacer-adjacent motif is a constraint in the guanine-cytosine-rich *Mycobacterium tuberculosis* genome (Zetsche et al., 2015; Li et al., 2018; Kaminski et al., 2021). Discrimination is also affected by mutation position, guide length, artificial mismatch placement, reaction temperature, target strand, and the enzyme-to-guide ratio (Zhang et al., 2023; Huang et al., 2021; Smith et al., 2021; Li et al., 2021). Primer and guide design must additionally consider lineage-associated polymorphisms: variation within primer-binding, PAM, protospacer, or guide-binding sites may alter amplification or recognition and cause allele dropout. Regional panels should therefore be screened against representative local genomic diversity and analytically revalidated after target updates rather than assuming that a guide design transfers unchanged across lineages.

Engineered Cas12a variants expand targetable protospacer-adjacent motifs and have enabled detection of otherwise constrained *rpsL* and *katG* mutations (Kleinstiver et al., 2019; Fuchs et al., 2022; Liu P. et al., 2022; Liu P. et al., 2025). Asymmetric amplification can generate single-stranded targets that reduce some protospacer-adjacent-motif constraints (Cao et al., 2023), as illustrated in Figure 4E. Miniature nucleases, including Cas12f systems formerly referred to as Cas14, offer alternative routes to target recognition (Harrington et al., 2018; Karvelis et al., 2020; Su et al., 2024). Following foundational Cas9 studies (Jinek et al., 2012), tuberculosis applications have included the MSPQC nucleic acid sensor (Huang J. et al., 2022). The broader FLASH method established Cas9-guided sequencing enrichment (Quan et al., 2019), and FLASH-TB subsequently adapted this strategy for direct-sputum resistance profiling (Tram et al., 2023). Other Cas9-based genotyping systems include CARP, which couples guide-directed cleavage to quantitative polymerase chain reaction for two *rpoB* variants, and ACURAT, which introduces targetable protospacer-adjacent motifs through specialized amplification to resolve multiple *rpoB* genotypes (Augustin and Agarwal, 2023; Zheng et al., 2024). Assays described in their source publications as Cas14a have also been applied to *rpoB*, *katG*, and *inhA* variants (Xiao et al., 2025; Wu G. et al., 2025). These strategies enlarge the design space, but broader molecular accessibility does not by itself increase drug-level coverage; coverage depends on how much of the clinically relevant resistance spectrum is represented by validated guides.

**FIGURE 4:**
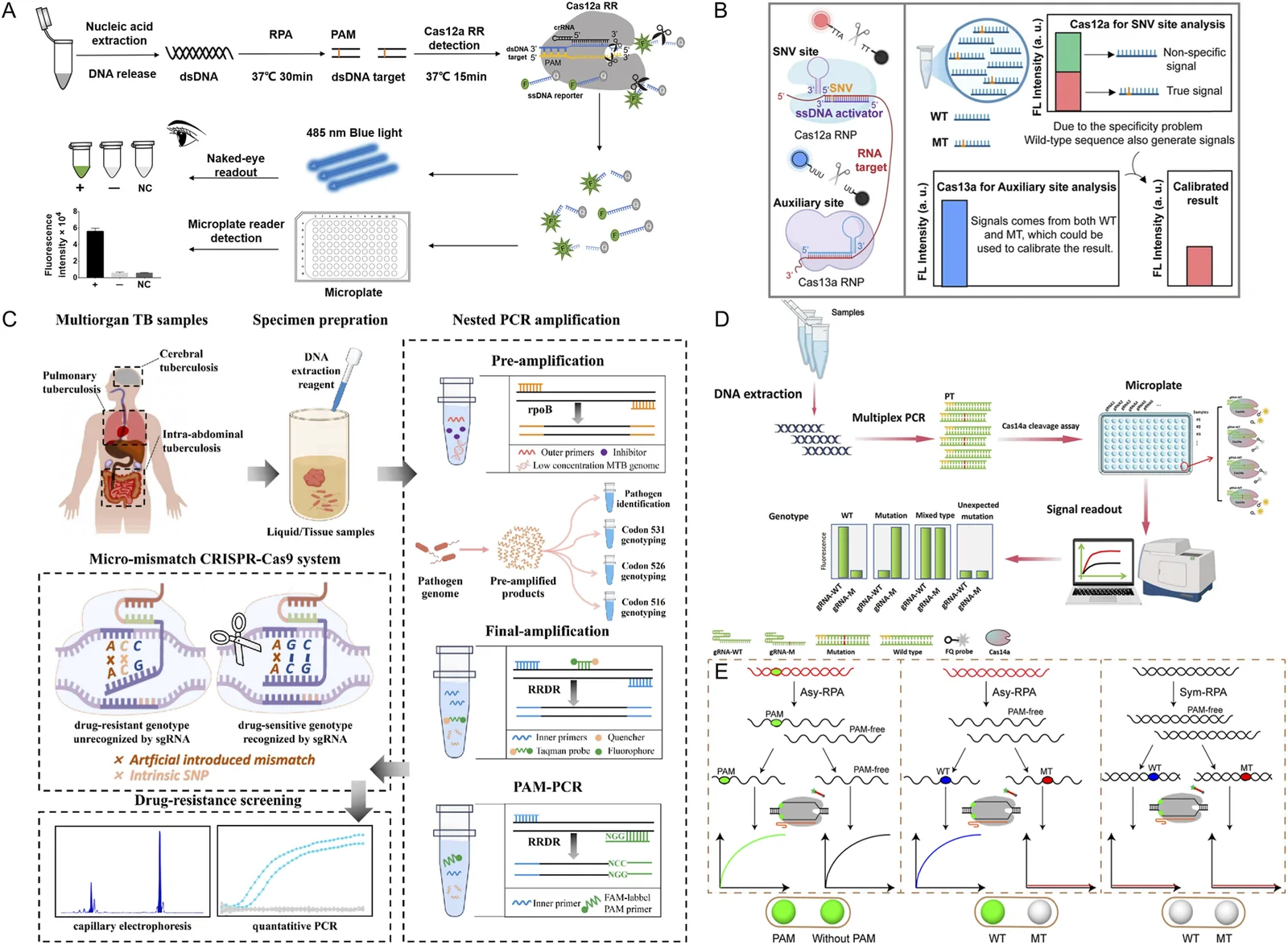
Representative molecular strategies for single-nucleotide-variant discrimination and resistance genotyping in CRISPR-based assays. **(A)** Engineered Cas12a RR-based assays can be coupled with RPA to detect PAM-constrained resistance-associated mutations and generate fluorescence or naked-eye readouts (Liu P. et al., 2022). Copyright © 2022 Liu et al.; distributed under the terms of the Creative Commons Attribution License (CC BY). **(B)** iCasdrop integrates Cas12a and Cas13a in a one-pot dual-channel system, using an auxiliary Cas13a signal to calibrate nonspecific wild-type background during single-nucleotide-variant detection. Reproduced with permission from Gao et al. (2025). Copyright © 2025 The Royal Society of Chemistry. **(C)** ACURAT combines nested PCR, PAM-PCR, and a micro-mismatch CRISPR–Cas9 system to resolve multiple *rpoB* rifampicin-resistance genotypes (Zheng et al., 2024). Copyright © 2024 The Author(s); published by Elsevier **(B)** V. under the Creative Commons Attribution 4.0 International License (CC BY 4.0). **(D)** Multiplex Cas14a-based workflows illustrate how wild-type, mutant, mixed-genotype, and unexpected-mutation signal patterns can be interpreted after multiplex PCR amplification (Xiao et al., 2025). Reproduced with permission from Xiao et al. (2025). Copyright © 2025 The Author(s); published by BMC. **(E)** Asymmetric RPA generates ssDNA products that activate Cas12a in a PAM-independent manner and can distinguish wild-type from rifampicin-resistant mutant templates. Adapted with permission from (Cao et al., 2023). Copyright © 2023 American Chemical Society. All required permissions for reproduced or adapted panels were obtained.

### 2.4 Wild-type suppression and preservation of mutant fractions

Resistance testing imposes stricter demands on amplification than pathogen detection because allele proportions carry diagnostic information. Preferential amplification of wild type can mask a minority resistant population, whereas uneven allele-specific priming can cause allele dropout, spurious allelic imbalance, or false-positive calls. Restriction-enzyme depletion of wild-type templates, allele-specific amplification, asymmetric amplification, artificial guide mismatches, and digital partitioning have been used to increase discrimination of low-frequency variants (Zhang et al., 2023; Huang et al., 2021; Liu P. et al., 2022; Liu P. et al., 2025; Cao et al., 2023; Wu et al., 2021). The dual Cas12a/Cas13a iCasdrop system provides a potentially transferable single-nucleotide-variant calibration strategy, but it has not established tuberculosis-specific clinical performance and does not constitute direct clinical validation for drug-resistant tuberculosis (Gao et al., 2025).

For conceptual clarity, we use three author-defined, non-consensus threshold categories; these are not proposed clinical cutoffs. The analytical detection threshold is the lowest mutant fraction at which a signal exceeds background under controlled conditions. The reproducible-reporting threshold is the lowest fraction detected consistently across runs, reagent lots, operators, and clinically relevant specimen backgrounds. The clinical-action threshold would require evidence that a specified fraction justifies treatment or confirmatory-testing action. Any mutant-fraction threshold is interpretable only when adequate total amplifiable *Mycobacterium tuberculosis* input is present (Metcalfe et al., 2017; Dreyer et al., 2020; Colman et al., 2015): at low copy number, stochastic molecular sampling can make a nominal minority fraction physically unobservable. This is why low-input samples are linked to the invalid or insufficient-input category rather than treated as valid negatives. Deep targeted sequencing offers broader mutation discovery and lineage context but depends on depth, error control, and bioinformatics; digital PCR provides sensitive quantification of prespecified alleles but remains targeted. CRISPR may provide rapid targeted discrimination, but amplification bias, guide selectivity, and template input must be reported with the fraction threshold.

### 2.5 Multiplex amplification, allele dropout, and panel-level coverage

Multiplexing is essential for drug-resistant tuberculosis because treatment decisions require combinations of loci and drugs. However, the nominal number of primers or guides is not equivalent to effective panel breadth. In multi-allele-specific polymerase chain reaction coupled to Cas12a, competition among ten *rpoB* primer sets caused partial amplification failure, requiring primer grouping and optimization of ratios, annealing temperature, and cycle number (Wu et al., 2024). Similar interactions may occur when amplification and guide reactions are combined in a single tube.

Multiplex capacity should therefore be reported at three levels: the number of primers or guides included, the number of targets that remain analytically valid when combined, and the proportion of resistant phenotypes expected to be covered in the intended population. To anchor the third level quantitatively without implying performance of an unbuilt CRISPR panel, we used published population-level upper bounds for molecular interrogation of the relevant regions. In the WHO-catalogue-derived dataset analyzed by Brankin et al. (2022), *rpoB* RRDR interrogation identified 9,135 of 9,869 phenotypically rifampicin-resistant isolates (92.6%; exact 95% CI 92.0–93.1). For isoniazid, the multicountry dataset of Dean et al. (2020) documented *katG* codon 315 and/or *inhA*-promoter mutations in 1,003 of 1,174 resistant isolates (85.4%; exact 95% CI 83.3–87.4); the incremental contribution of *fabG1* cannot be added independently without co-occurrence data. For fluoroquinolones, Brankin et al. estimated the upper-bound sensitivity of GenoType MTBDRsl v2 wild-type-probe interrogation across *gyrA* codons 85–96 and *gyrB* codons 497–502 at 2,648/3,125 for levofloxacin (84.7%; exact 95% CI 83.4–86.0) and 1,658/1,880 for moxifloxacin (88.2%; exact 95% CI 86.6–89.6) (Brankin et al., 2022). These fluoroquinolone values describe a region-interrogating comparator and do not imply that an allele-specific CRISPR panel interrogates the full *gyrA*/*gyrB* regions. They are region-level comparator ceilings, not the measured coverage of the proposed CRISPR architecture. Because the proposed architecture does not specify a finalized guide/allele list, an exact panel-level coverage percentage is not estimable at present; once guide content is fixed, panel coverage should be recalculated from the exact targeted alleles in a prespecified genotype-phenotype dataset. A finite allele-specific guide set can only equal or fall below the relevant region-level ceiling because of guide content, lineage variation, multiplex dropout, molecular input, and readout thresholds (Supplementary Table S2).

### 2.6 Direct-specimen processing and CRISPR-assisted enrichment

In direct sputum, *Mycobacterium tuberculosis* DNA is diluted by human and microbial DNA, protected by a lipid-rich cell wall, and exposed to amplification inhibitors. Sample preparation must release sufficient target while preserving minority alleles. Rapid CRISPR readout assays, integrated sample-to-result devices, and CRISPR-assisted sequencing enrichment address different aspects of this problem. Compact readout assays prioritize a limited number of actionable variants and short turnaround time; integrated devices prioritize containment and workflow automation; enrichment strategies prioritize broader coverage and improved sequencing depth.

FLASH-TB illustrates the enrichment approach. A Cas9 guide set targeting identification regions and drug-resistance genes increased the fraction and depth of *Mycobacterium tuberculosis* reads in direct sputum, improving the proportion of specimens for which resistance prediction was possible (Tram et al., 2023). A rolling-circle-amplification-Cas12a study used padlock probes and a one-tube cassette to screen four rifampicin-resistance-associated SNVs. The full paper confirms evaluation of 16 sputum or bronchoalveolar-lavage specimens against Sanger sequencing, with source-reported sensitivity of 100% and specificity of 83.3%; however, its displayed contingency table contains internally inconsistent labels/totals, so exact clinical counts should be interpreted cautiously (Wu Y. et al., 2025). A thin-film microfluidic system integrated chemical lysis, alcohol-free nucleic acid purification, multiplex asymmetric PCR, PAM-independent Cas12a analysis, and a dual-signal readout for seven *rpoB* RRDR mutations. It completed extraction (18 min), amplification (45 min), and CRISPR analysis (15 min) in 78 min and evaluated 28 clinical specimens against Sanger sequencing, but the report does not clearly describe specimen composition or provide phenotype-level contingency data (Cheng et al., 2026). These approaches address different constraints and should not be judged by the same endpoint.

### 2.7 Closed workflows, internal controls, and readout interpretation

The readout directly affects clinical interpretation. Fluorescence supports kinetic analysis and predefined thresholds, whereas lateral-flow and colorimetric formats simplify decentralized use but can make weak bands difficult to interpret consistently (Khosla et al., 2025; Patchsung et al., 2020; Posthuma-Trumpie et al., 2009; Zhang et al., 2021; Liu et al., 2025c). Electrochemical, field-effect transistor, Raman, paper-based, and digital-partitioning readouts offer quantification or miniaturization, although most remain at methodological or small-cohort stages (Dai et al., 2019; Yin et al., 2022; Hajian et al., 2019; Yin et al., 2021; Zhang et al., 2024).

A one-pot biochemical reaction is not necessarily a closed sample-to-result system. Reports need to state whether lysis, nucleic-acid transfer, amplification, CRISPR detection, and readout occur without opening the reaction vessel. This distinction matters in resistance testing because amplicon contamination can produce a false resistance call and alter therapy. The rolling-circle-amplification-Cas12a cassette integrated ligation, amplification, and CRISPR readout in one tube but did not integrate automated primary-specimen extraction (Wu Y. et al., 2025). The thin-film microfluidic study integrated extraction, amplification, CRISPR genotyping, and readout in a sealed device, but its small clinical evaluation and restricted *rpoB* coverage do not support comprehensive exclusion of rifampicin resistance (Cheng et al., 2026). Internal controls should separately address extraction/lysis, amplifiable *Mycobacterium tuberculosis* input, and reaction inhibition. IS6110 is useful for sensitive MTBC detection but is not an ideal quantitative input denominator because its copy number varies across MTBC strains; a validated single-copy target is preferable when allele fraction is clinically relevant. The main engineering bottlenecks and evidence requirements are summarized in Table 3.

**TABLE 3:** Engineering bottlenecks, solution modules, and diagnostic claims at risk.

Bottleneck	Molecular consequence	Potential solution	Claim at risk	Required evidence
Protospacer-adjacent motif restriction	Clinically relevant variants cannot be targeted	Engineered Cas variants; artificial protospacer-adjacent motif amplification; single-stranded recognition	Mutation coverage	Variant-specific analytical validation
Wild-type background	Minor resistant alleles are masked	Wild-type depletion; allele-specific amplification; digital partitioning; ratio calibration	Mutation detection	Mutant-fraction series in relevant backgrounds
Multiplex primer competition	Allele dropout or uneven amplification	Primer grouping; ratio optimization; partitioned reactions	Panel-level sensitivity	Combined-panel interference testing
Low bacillary load and inhibitors	Invalid or biased direct-specimen results	Improved lysis; concentration; enrichment; internal controls	Specimen validity	Bacillary-load-stratified direct-specimen validation
Open-tube transfer	Amplicon carryover and false resistance calls	Closed reaction architecture; contamination controls	Specificity and traceability	Carryover challenge and invalid-rate testing
Subjective visual readout	Inconsistent weak-positive interpretation	Quantitative fluorescence; digital imaging; electrochemical or droplet readout	Report category	Prespecified cutoffs and inter-operator agreement
Incomplete regional coverage	Phenotypic resistance is missed	Updatable regional panels informed by sequencing and phenotypic surveillance	Drug-level sensitivity	Population-specific coverage analysis and revalidation

## 3 Mutation-to-drug evidence mapping

The ability to redesign CRISPR guides is clinically useful only when the selected targets are relevant to treatment. Target priority should reflect therapeutic actionability, the extent to which clinically relevant resistance variants are concentrated within a limited number of loci, the stability of genotype–phenotype associations, regional prevalence, and whether a positive result would change a regimen or prompt confirmatory testing (Wells et al., 2013; Vanino et al., 2023; Allix-Béguec et al., 2018; Farhat et al., 2019). Ease of guide design should not, by itself, determine panel inclusion.

Interpretation should be anchored to the formal evidence categories in the World Health Organization mutation catalogue and related evidence tiers (Miotto et al., 2017; Walker et al., 2022; World Health Organizatio n, 2023). In this review, WHO Group 1 (associated with resistance) provides the clearest basis for a categorical resistance rule-in. Group 2 variants (associated with resistance - interim) are reported explicitly as interim-evidence variants rather than merged unqualified with Group 1. Group 3 variants are variants of uncertain significance and do not support an unqualified resistant call; they require contextual interpretation and, where clinically important, orthogonal confirmation. The phrase “well-validated resistance-associated variant” is therefore used primarily for Group 1 or equivalently validated evidence and is not an additional WHO category. Modern amino-acid numbering is used, with legacy *rpoB* numbering provided parenthetically where clinically familiar. A positive result supports resistance only for the relevant drug and within the population and analytical context in which the association and assay have been validated.

### 3.1 Rifampicin: a model for high-value rule-in testing

Rifampicin resistance is a core entry point to the multidrug- or rifampicin-resistant tuberculosis pathway and is the best-characterized molecular target for resistance testing (Boehme et al., 2010; Telenti et al., 1993). The rifampicin resistance-determining region (RRDR) is defined here as *rpoB* codons 426–452 in modern *Mycobacterium tuberculosis* numbering (507–533 in legacy numbering). Common resistance-associated variants include p.Ser450Leu (legacy S531L), p.His445 substitutions (legacy H526), and p.Asp435 substitutions (legacy D516) (Rostamian et al., 2023; CRyPTIC Consortium, 2022; Molodtsov et al., 2017). This concentration makes the RRDR suitable for compact rule-in panels, while the residual extra-RRDR resistance fraction limits rule-out interpretation.

CRISPR studies address different aspects of the *rpoB* problem. CARP detects two selected variants, p.Ser450Trp (legacy S531W) and p.His445Cys (legacy H526C), through Cas9 cleavage and quantitative polymerase chain reaction and has so far been evaluated primarily at the analytical or methodological level (Augustin and Agarwal, 2023). ACURAT combines nested polymerase chain reaction, PAM-introducing polymerase chain reaction (PAM-PCR), and micro-mismatch Cas9 guides to distinguish 15 genotypes at modern codons 435, 445, 450, and 452 (legacy 516, 526, 531, and 533) (Figure 4C); however, the study did not establish direct-specimen diagnostic accuracy (Zheng et al., 2024). A dedicated Cas14a study used eight guides for common rifampicin resistance-determining-region variants and achieved complete concordance with sequencing across 16 cultured clinical isolates; it relied on extracted isolate DNA, asymmetric polymerase chain reaction, and separate Cas14a reactions rather than direct sputum testing (Wu G. et al., 2025).

Beyond single-variant assays, several studies have expanded either panel breadth or workflow integration. Multi-allele-specific polymerase chain reaction coupled to Cas12a evaluated ten *rpoB* variants in respiratory specimens but required primer grouping because of multiplex competition (Wu et al., 2024). A rolling-circle-amplification-Cas12a cassette screened four single-nucleotide variants in one tube and reported 100% sensitivity but 83.3% specificity against sequencing, showing that physical integration does not remove the need to assess specificity for the assembled panel (Wu Y. et al., 2025). The thin-film system integrated extraction, multiplex asymmetric polymerase chain reaction, and PAM-independent CRISPR analysis for seven *rpoB* mutations in a 28-sample evaluation that was concordant with Sanger sequencing (Cheng et al., 2026), whereas FLASH-TB broadened direct-sputum resistance profiling through Cas9-guided sequencing enrichment (Tram et al., 2023).

The *rpoB* literature also shows why mutation-level performance must be distinguished from drug-level sensitivity. Detecting one common variant can provide strong rule-in evidence but will miss resistant phenotypes caused by other *rpoB* variants. An RPA-Cas12a assay for *rpoB* p.Leu378Arg illustrates technical programmability toward a noncanonical target (Yang et al., 2026). However, p.Leu378Arg is classified in the 2023 WHO catalogue as Group 3 (uncertain significance) and should not be used as an established categorical resistance marker (World Health Organizatio n, 2023). Catalogued resistance-associated variants outside the conventional RRDR include p.Val170Phe and p.Ile491Phe, among others, and may be epidemiologically important in particular settings (World Health Organizatio n, 2023; Barilar et al., 2024). A negative RRDR or compact exact-variant panel therefore applies only within its validated target boundary.

### 3.2 Isoniazid: distinct mechanisms require distinct interpretations

Isoniazid remains a core first-line drug, and isoniazid resistance can undermine standard treatment even when rifampicin remains active (Dean et al., 2020; Diacon et al., 2020; Gegia et al., 2017). Coexisting rifampicin and isoniazid resistance defines multidrug-resistant tuberculosis, while isoniazid-resistant, rifampicin-susceptible tuberculosis requires a different treatment pathway. Rifampicin testing therefore cannot substitute for isoniazid testing.

The genetic basis of isoniazid resistance is more heterogeneous than that of rifampicin resistance (Seifert et al., 2015; Valafar, 2021). *katG* p.Ser315Thr is common and generally carries strong rule-in value, whereas *inhA* promoter variants have different implications for resistance level and possible ethionamide cross-resistance (Lempens et al., 2018; Vilchèze and Jacobs, 2014). Auxiliary loci such as *fabG1* and *ahpC* may contribute but require more cautious interpretation.

Isoniazid-related CRISPR findings can be reported in three clinically distinct categories: a validated *katG* variant with strong resistance evidence, a validated *inhA* promoter variant with explicit cross-resistance interpretation, and no targeted isoniazid-resistance variant detected. The engineered Cas12a-RR study targeting *katG* p.Ser315Thr used restriction-enzyme depletion of wild type, detected an approximately 1% mutant fraction in controlled mixtures, and evaluated 42 cultured clinical isolates, including 27 *katG* c.944G>C-positive and 15 mutation-negative isolates (Liu P. et al., 2025). Fluorescence and lateral-flow readouts separated the targeted mutant and wild-type isolate groups within an approximately 60-min post-extraction workflow. These results provide useful isolate-level evidence for the specified variant, but they do not constitute direct-specimen validation or establish sensitivity for all phenotypic isoniazid resistance.

The Cas9 electrochemical sensor for *katG* codon 315 used designed DNA targets and remains analytical biosensor evidence rather than a clinically validated isoniazid-resistance test (Wulandari et al., 2025). The direct-sputum Cas14a platform included *katG* and the *inhA* promoter, whereas the AaCas12b study covered only *katG* c.944G>C. Its signal with the alternative resistance-associated *katG* p.Ser315Asn (c.944G>A) is better described as limited allele specificity rather than clinically irrelevant cross-reactivity (Xiao et al., 2025; Hu et al., 2026). These findings underline the need to report exact-variant specificity separately from phenotype sensitivity. A compact first-line rule-in layer can prioritize *katG* codon 315 together with the clinically relevant *inhA*/*fabG1* promoter region, with additional loci selected according to regional evidence.

### 3.3 Fluoroquinolones: regimen-defining resistance detection and pre-extensively drug-resistant tuberculosis classification

Fluoroquinolones are central to all-oral regimens for multidrug- or rifampicin-resistant tuberculosis and to the definition of pre-extensively drug-resistant tuberculosis (Viney et al., 2021; Sinha et al., 2023; Pranger et al., 2019; Choi et al., 2023; Kempker et al., 2015; Bastard et al., 2018). The principal resistance-associated region is the quinolone resistance-determining region of *gyrA*, with recurrent variants at codons 90, 91, and 94; selected *gyrB* variants may also contribute (Von Groll et al., 2009; Farhat et al., 2016; Pantel et al., 2011).

In 75 clinical isolates, a Cas13a assay covering seven common *gyrA* variants achieved 91.4% sensitivity and 100% specificity against phenotypic testing (Bai et al., 2022). Resistant isolates without the targeted variants remained negative. The assay therefore supports rule-in of selected fluoroquinolone resistance in isolates, but direct-sputum performance and coverage of *gyrB* or other mechanisms remain untested.

Detection of a validated *gyrA* or *gyrB* variant classified as associated with resistance can support rapid fluoroquinolone-resistance rule-in. In a patient with confirmed multidrug- or rifampicin-resistant tuberculosis, that result can contribute to pre-extensively drug-resistant tuberculosis classification. The absence of targeted *gyrA* or *gyrB* variants should not, by itself, be interpreted as indicating a low probability of fluoroquinolone resistance and does not establish fluoroquinolone susceptibility. When coverage is incomplete, specimen input is low, or clinical suspicion remains high, targeted sequencing, whole-genome sequencing, or phenotypic confirmation is appropriate.

### 3.4 Region- and regimen-dependent targets

The therapeutic role of streptomycin and second-line injectable drugs has declined in many settings as all-oral regimens have expanded, but their resistance markers retain regional, historical, and epidemiological relevance (Sinha et al., 2023; Mirzayev et al., 2021; Cegielski et al., 2021; Dean et al., 2022; Wang et al., 2019; Georghiou et al., 2012). Targets such as *rpsL*, *rrs*, and the *eis* promoter may still influence treatment where these drugs remain in use or in patients with substantial prior exposure.

CRISPR studies targeting *rpsL* demonstrate the technical feasibility of detecting low-frequency variants and expanding beyond rifampicin and isoniazid (Figure 4A) (Liu P. et al., 2022). These loci are better treated as regional or regimen-dependent extensions than as universal core targets. Their inclusion should be justified by local drug use, mutation prevalence, and a clear link to treatment selection or confirmatory testing.

### 3.5 Targets that define the boundary of compact CRISPR panels

Clinical importance does not necessarily imply suitability for a compact hotspot panel. Pyrazinamide resistance is associated with diverse variants distributed across *pncA* and its promoter (Whitfield et al., 2022; Cheng et al., 2000; Ramirez-Busby and Valafar, 2015; Yadon et al., 2017). More fundamentally, an allele-specific CRISPR assay interrogates prespecified variants and cannot comprehensively exclude unenumerated substitutions, indels, frameshifts, or other loss-of-function changes. Region-level sequencing addresses a different and broader diagnostic claim (Georghiou et al., 2025; Phelan et al., 2016). Wild-type-probe or dropout approaches can likewise interrogate sequence deviation across a predefined region, but represent a distinct assay architecture (Brankin et al., 2022). Species context also matters: *Mycobacterium bovis* is intrinsically pyrazinamide resistant and is characteristically associated with *pncA* p.His57Asp, so species-level information should be considered in the identification layer when pyrazinamide interpretation is relevant. A small exact-variant guide set therefore has limited pyrazinamide rule-out value, whereas an exhaustive guide set may approach the complexity of targeted sequencing.

Ethambutol resistance often involves *embB*, but genotype-phenotype associations are variant-specific and may depend on minimum inhibitory concentration distributions and testing conditions (Starks et al., 2009; Plinke et al., 2010). Ethionamide resistance involves *ethA*, the *inhA* promoter, and other mechanisms, including diverse loss-of-function variants that are not comprehensively captured by a finite set of allele-specific guides (Vilchèze and Jacobs, 2014; DeBarber et al., 2000). Together, *pncA*, *embB*, and *ethA* illustrate a practical boundary for compact CRISPR panels: allele-specific assays are most useful when validated variants are concentrated and actionable, whereas dispersed or heterogeneous mechanisms are better handled by region-level sequencing, CRISPR-assisted sequencing enrichment, or phenotypic testing where appropriate.

### 3.6 New and repurposed drugs: diagnostic evidence versus mutation discovery

Bedaquiline, clofazimine, linezolid, delamanid, and pretomanid are increasingly important components of treatment for drug-resistant tuberculosis (Günther et al., 2024; Nyang’wa et al., 2022; Conradie et al., 2022; Conradie et al., 2020). Resistance to bedaquiline and clofazimine can involve *Rv0678*, *atpE*, *pepQ*, and related loci; linezolid resistance can involve *rrl* and *rplC*; and nitroimidazole resistance can involve *ddn*, *fgd1*, and *fbi* genes (World Health Organizatio n, 2023; Sonnenkalb et al., 2023; Liu Y. et al., 2022; Rifat et al., 2021). Many clinically relevant changes in *Rv0678*, *ethA*, *ddn*, and related genes are heterogeneous or loss-of-function variants. An allele-specific CRISPR panel detects only enumerated variants and therefore cannot comprehensively exclude these mechanisms. Region-level targeted sequencing or whole-genome sequencing is better suited when detection of previously unenumerated substitutions, indels, or truncating variants is required.

Diagnostic evidence should be separated from mutation-discovery evidence. CAMPER used CRISPR-guided mutagenesis to functionally support an existing association between *atpE* p.Ile66Met (*Mycobacterium tuberculosis* numbering) and bedaquiline resistance rather than identify a new clinically validated diagnostic variant (Feng et al., 2022). This work is relevant to mutation discovery and functional validation but does not constitute patient-sample diagnostic accuracy evidence. Candidate variants should enter clinical panels only after independent genotype-phenotype confirmation, assessment of prevalence and specificity, and demonstration of treatment relevance. In the near term, complex resistance to newer drugs is better addressed by targeted or whole-genome sequencing, CRISPR-assisted enrichment, and, where technically validated and clinically interpretable, phenotypic drug susceptibility testing.

### 3.7 A layered and updatable panel architecture

CRISPR-based panels should be layered by intended use rather than designed as exhaustive lists of detectable loci. The author-proposed core resistance-detection layer combines an MTBC identification signal with separate extraction, single-copy quantitative-input (where allele fractions are interpreted), and reaction controls; *rpoB* RRDR targets; *katG* codon 315; and the clinically relevant *inhA*/*fabG1* promoter region. Its intended use is rapid rule-in of selected rifampicin- and isoniazid-resistance variants and early entry into an expanded diagnostic pathway. Population-level biological coverage remains incomplete even for these high-yield regions (Supplementary Table S2), so a compact negative result cannot substitute for broader WHO-recommended resistance testing when clinically indicated.

A regimen-defining expansion would cover the *gyrA* quinolone resistance-determining region and selected drug-specific WHO-supported *gyrB* variants, with regional prevalence and drug-specific evidence considered explicitly. A reference or sequencing-assisted layer would address dispersed and complex mechanisms involving *pncA*, *Rv0678*, nitroimidazole-activation genes, *rrl*, and *rplC*. Each layer needs a predefined intended use, positive-call rule, negative-result boundary, internal-control requirement, confirmatory pathway, and update process. Guide additions should be based on mutation catalogues, regional sequencing and phenotypic surveillance, and treatment relevance, followed by analytical and direct-specimen revalidation. A recent *in silico* study designed candidate guide RNAs for selected resistance variants prevalent in Indian genomes, but these candidates still require laboratory and clinical validation (Mittal et al., 2026). The proposed architecture is summarized in Table 4 and its population-level coverage benchmarks in Supplementary Table S2.

**TABLE 4:** Layered, treatment-oriented architecture for CRISPR-enabled drug-resistant tuberculosis testing.

Layer	Intended use	Example targets	Allowed interpretation	Interpretation not supported	Recommended follow-up
Assay validity, pathogen input, and process controls	Establish interpretable *Mycobacterium tuberculosis* input and verify extraction and reaction validity	MTBC detection target (e.g., IS6110/IS1081); validated single-copy quantitative input target when allele fraction is interpreted; separate extraction and reaction controls	Valid, invalid, or insufficient-input resistance result	Do not infer that a control-failed or low-input negative result excludes tuberculosis or resistance	Repeat testing, Xpert, culture, or sequencing
Core RIF/INH rule-in	Rapidly identify common, well-validated first-line resistance variants	*rpoB* RRDR; *katG* codon 315; clinically relevant *inhA*/*fabG1* promoter region	Detection of a targeted rifampicin- or isoniazid-resistance-associated variant	Do not infer that a negative panel proves susceptibility or excludes multidrug resistance	Xpert MTB/XDR, line-probe assay, sequencing, or phenotypic drug susceptibility testing where technically validated
FQ expansion	Rapid regimen-defining fluoroquinolone rule-in	*gyrA* QRDR; selected drug-specific WHO-supported *gyrB* variants	Detection of a validated *gyrA* or *gyrB* resistance-associated variant supports fluoroquinolone-resistance inference and, after multidrug/rifampicin resistance is established, pre-extensively drug-resistant tuberculosis classification	Do not infer that a negative result indicates low risk or that a fluoroquinolone result alone establishes extensively drug-resistant tuberculosis	Targeted or whole-genome sequencing and/or phenotypic drug susceptibility testing where technically validated
Regional and regimen-dependent expansion	Match local variants and drug use	*rpsL*, *rrs*, *eis*, selected regional *rpoB*, *gyrA*, *gyrB*, *embB*	Population-specific mutation detected with stated evidence tier	Do not generalize beyond the validated population	Regional sequencing/phenotypic surveillance and periodic revalidation
Sequence-assisted complex-resistance layer	Resolve dispersed, rare, or newer-drug mechanisms	*pncA*, *Rv0678*, *atpE*, *pepQ*, *rrl*, *rplC*, *ddn*, *fgd1*, *fbi* genes	A WHO-catalogued or otherwise well-validated variant may be interpreted in the relevant clinical context	Do not infer that a compact panel excludes all resistance to the drug	CRISPR-enriched sequencing, targeted sequencing, whole-genome sequencing, or phenotypic drug susceptibility testing where technically validated

Abbreviations: FQ, fluoroquinolone; INH, isoniazid; MTBC, *mycobacterium tuberculosis* complex; RIF, rifampicin; RRDR, rifampicin resistance-determining region.

## 4 Direct-specimen readiness, heteroresistance, and multiplex profiling

### 4.1 Direct sputum evidence

Sputum remains the principal specimen for pulmonary tuberculosis, but mucus, host cells, nontarget DNA, inhibitors, and the lipid-rich bacillary cell wall complicate direct molecular testing (Steingart et al., 2014; Votintseva et al., 2017; Theron et al., 2011). For resistance testing, sample preparation must not only release sufficient nucleic acid but also preserve the relative allele fractions of wild-type and resistant populations. Studies should report input volume, bacillary-load category, extraction or lysis control, invalid rate, and whether allele fractions are retained after amplification.

Although limited, the available direct-specimen evidence demonstrates technical feasibility. The multiplex Cas14a study evaluated 60 cultured isolates and 55 smear-positive, culture-positive sputum specimens (Xiao et al., 2025). In the isolate arm, 39 of 42 rifampicin-resistant isolates were detected, which is 92.9%, whereas the source reports 93.3% (95% CI 80.0%–98.5%); this arithmetic discrepancy is retained as a source-data inconsistency. In sputum, it detected rifampicin resistance in 10 of 12 phenotypically resistant specimens (83.3%; exact 95% CI 51.6%–97.9%) and isoniazid resistance in all 11 resistant specimens (100%; 95% CI 71.5%–100%). The source article reports 85.7% for the same rifampicin count, an internal numerical inconsistency. Both missed *rpoB* variants, p.Asp435Tyr and p.Leu452Pro, lie within the RRDR; because p.Asp435Tyr was nominally included in the assay design, that miss cannot confidently be attributed to target coverage and may represent analytical/interpretive failure or another source-data inconsistency, whereas p.Leu452Pro was not covered by the guide set. The workflow required multiplex PCR followed by transfer into separate Cas reactions and took approximately 2.5 h including extraction (Figure 4D). Because the cohort was restricted to smear-positive, culture-positive specimens, performance in paucibacillary sputum remains unknown.

The AaCas12b study evaluated 48 residual, pretreated sputum or bronchoalveolar-lavage specimens and illustrates why exact-mutation and drug-phenotype performance must be separated (Hu et al., 2026). Against sequencing, fluorescence detection of *rpoB* c.1349C>T achieved 94.1% sensitivity (16/17; exact 95% CI 71.3%–99.9%) and 100% specificity (31/31; 88.8%–100%), whereas *katG* c.944G>C achieved 94.7% sensitivity (18/19; 74.0%–99.9%) and 93.1% specificity (27/29; 77.2%–99.2%). Phenotype-level fluorescence sensitivity was lower (66.7% for rifampicin resistance and 79.2% for isoniazid resistance) because one variant per drug was targeted. Lateral-flow sensitivities differed from fluorescence despite identical molecular targets, illustrating that readout thresholds and weak-signal classification are part of the diagnostic test rather than neutral display choices. The reported 30–35-min assay time excludes approximately 40 min of specimen preparation.

Other direct-specimen studies remain small. Multi-AS-PCR-Cas12a evaluated 24 sputum or bronchoalveolar-lavage specimens (9 rifampicin-resistant and 15 rifampicin-sensitive by sequencing), achieving 100% sensitivity (9/9; exact 95% CI 66.4%–100%) and 93.3% specificity (14/15; 68.1%–99.8%), but ten allele-specific primers could not be retained as one balanced mixture and required C4/C6 grouping (Wu et al., 2024). The one-tube RCA-Cas12a study evaluated 16 sputum/BALF specimens; its reported 100% sensitivity and 83.3% specificity are compatible with 10/10 and 5/6 classifications, respectively, but the published contingency-table labels/totals are internally inconsistent and should be interpreted cautiously (Wu Y. et al., 2025). The thin-film microfluidic system tested 28 clinical specimens and reported full concordance with Sanger sequencing, but specimen composition, phenotype-level coverage, and class-specific contingency counts were not sufficiently described for an independent 95% confidence interval (Cheng et al., 2026). Additional isolate-level evidence includes a Cas9n/Cas9 self-priming assay targeting the *rpoB* RRDR: 20 cultured isolates (6 rifampicin-resistant, 14 susceptible) were concordant with phenotypic DST and PCR, corresponding to exact 95% CIs of 54.1%–100% and 76.8%–100% for sensitivity and specificity, respectively; however, the resistant mutation spectrum was not enumerated and no direct specimens were tested (Gan et al., 2026). No study in this evidence set provides prospective multicenter or treatment-outcome-linked validation.

### 4.2 Transferable specimen-engineering evidence

CRISPR-based tuberculosis studies have evaluated proof-of-concept detection across respiratory specimens, cerebrospinal fluid, pediatric stool, blood- or plasma-derived targets, and tongue swabs (Ai et al., 2019; Sam et al., 2021; Peng et al., 2024; Huang et al., 2025; Bell et al., 2025; Youngquist et al., 2025; Thakku et al., 2023; Huang Z. et al., 2022; Liu et al., 2025a; Yu et al., 2026). A peer-reviewed one-pot biplex RPA-Cas12a assay targeting IS1081 and IS6110 further demonstrated 30-min MTBC pathogen detection from clinical tongue swabs with an analytical sensitivity of 1.1 genomes per 15-µL reaction (Cantera et al., 2026). Because it does not interrogate resistance variants, it is included only as transferable specimen-engineering and closed-workflow evidence. Recent crRNA-control and paperfluidic studies likewise inform programmable reaction control and portable readout (Wang et al., 2026; Thepbut et al., 2026) but do not establish resistance-associated allele-fraction fidelity in the same matrices.

Bronchoalveolar lavage fluid may be relevant in patients unable to produce sputum, whereas cerebrospinal fluid or cell-free DNA from pleural fluid may support microbiological confirmation in paucibacillary disease. Resistance testing in these matrices requires independent validation because fragmentation, low target abundance, and matrix-specific inhibition may affect variant coverage and minority-allele fidelity. Until such studies are available, these specimen types should be described as transferable engineering models rather than established direct-specimen evidence for drug-resistant tuberculosis.

### 4.3 Workflow simplification and contamination control

Many CRISPR assays depend on amplification. Opening a tube after amplification and transferring product into a detection reaction poses a substantial risk of amplicon carryover. In resistance testing, contamination can generate not only false-positive pathogen detection but also a false resistance call with immediate treatment consequences. The terms one-pot, one-tube, closed-tube, and sample-to-result should therefore be used precisely rather than interchangeably. The Xiao Cas14a and Hu AaCas12b workflows still required separate preparation and/or post-amplification handling (Xiao et al., 2025; Hu et al., 2026). The rolling-circle-amplification-Cas12a cassette enclosed ligation, rolling-circle amplification, and Cas12a detection but did not integrate primary-specimen extraction (Wu Y. et al., 2025), whereas the thin-film microfluidic study integrated extraction, amplification, CRISPR analysis, and readout in a sealed automated device (Cheng et al., 2026).

An integrated workflow needs to address specimen inactivation, lysis, nucleic-acid release, amplification, CRISPR detection, objective readout, and waste containment. For the RCA-Cas12a study, the reported 10 fM analytical limit in a 20-µL reaction corresponds to approximately 1.2 × 10^5^ target copies per reaction; this unit-normalized value is shown in Table 1 and should be interpreted alongside the study’s small, internally inconsistent clinical dataset (Wu Y. et al., 2025). The RCA-Cas12a cassette shows how several molecular steps can be consolidated in one tube, but its limited four-variant coverage, internally inconsistent clinical summary table, and lack of integrated primary-specimen extraction mean that workflow integration and clinical reliability must be assessed separately (Wu Y. et al., 2025). The thin-film system is among the most complete sample-to-result demonstrations to date, but its seven-variant *rpoB* panel and 28-specimen evaluation do not establish phenotype-level sensitivity, programmatic invalid rates, or multicenter reproducibility (Cheng et al., 2026). Its source-reported 0.1 copies/µL limit at a 20% mutant fraction is retained as reported but is molecular-sampling dependent: if interpreted as total template concentration, the expected mutant copy number is only one-fifth of the total sampled molecules, so reaction volume and the denominator used by the authors are essential for interpretation. Analytical limits are therefore converted to copies per reaction only when the source provides an unambiguous input volume; otherwise original units are retained and the conversion is marked not derivable.

### 4.4 Heteroresistance, low-frequency variants, and mixed infection

Heteroresistance is a major challenge because drug-susceptible and drug-resistant populations can coexist in one specimen (Folkvardsen et al., 2013; Operario et al., 2017). The clinical importance of a minority resistant population depends on its fraction, the drug and regimen, prior treatment, the risk of resistance amplification, and the interpretive threshold used (Ängeby et al., 2012). Analytical sensitivity alone cannot determine a treatment decision.

Tuberculosis-specific strategies include engineered Cas12a detection of low-frequency *rpsL* variants, wild-type depletion for *katG*, asymmetric amplification, and enrichment sequencing (Liu P. et al., 2022; Liu P. et al., 2025; Cao et al., 2023; Tram et al., 2023). iCasdrop uses an auxiliary Cas13a signal to calibrate nonspecific wild-type background during Cas12a-based single-nucleotide-variant detection (Figure 4B), but its performance for tuberculosis drug-resistance testing has not been established in direct clinical specimens (Gao et al., 2025). These methods show that minority variants can be detected under controlled conditions, but reported fractions must be interpreted together with absolute amplifiable MTBC input. Deep targeted sequencing can detect a broader spectrum of minority variants and provide lineage context, whereas digital PCR offers sensitive quantification of prespecified alleles; neither is directly interchangeable with a CRISPR signal threshold. A low-frequency CRISPR signal also cannot by itself distinguish within-host heteroresistance from mixed infection with different strains. Deep sequencing, lineage markers, repeat sampling, or paired culture analysis may therefore be required.

Low-frequency reporting should apply the three conceptual threshold categories defined above and should be withheld when total MTBC input is insufficient to support the claimed mutant fraction. These categories are not standardized tuberculosis clinical cutoffs. An unvalidated low-frequency signal should not trigger a categorical treatment decision without repeat testing or orthogonal confirmation.

### 4.5 Multiplexing breadth versus interpretive complexity

Clinical management requires multilocus and multidrug interpretation, but existing multiplex studies vary in both breadth and intended use. The Xiao Cas14a and Hu AaCas12b assays combined *Mycobacterium tuberculosis* identification with selected rifampicin- and isoniazid-associated variants, whereas the Wu Cas14a, CARP, ACURAT, multi-allele-specific polymerase chain reaction-Cas12a, and Cas12a-RR studies focused on increasing variant coverage or improving single-base discrimination (Liu P. et al., 2025; Augustin and Agarwal, 2023; Zheng et al., 2024; Xiao et al., 2025; Wu G. et al., 2025; Wu et al., 2024; Hu et al., 2026). More integrated systems include the four-variant rolling-circle-amplification-Cas12a cassette and the seven-variant thin-film device; FLASH-TB instead broadens coverage through sequencing enrichment (Tram et al., 2023; Wu Y. et al., 2025; Cheng et al., 2026).

As the number of targets increases, the limiting factor may shift from Cas recognition to amplification balance, guide competition, channel capacity, and result interpretation. Multiplex studies need to report dropout by locus, cross-target interference, invalid patterns, and the proportion of phenotypic resistance covered by the complete panel. Parallel partitioning warrants evaluation as a potentially more robust alternative to combining all targets in a single nominally multiplexed reaction.

## 5 Clinical reporting, benchmarking, and implementation

### 5.1 Claim-specific performance evaluation

Applying the four claim levels defined above, assay validity requires successful extraction or lysis controls, adequate *Mycobacterium tuberculosis* input, and successful reaction controls. Mutation-level performance should be compared with sequencing for the exact targeted variants. Drug-level performance should be assessed against an accepted mutation catalogue and, where technically validated and clinically interpretable for the drug under study, phenotypic drug susceptibility testing. Decision impact should measure whether the result changes time to an effective regimen, referral, or confirmatory testing. These evaluations are consistent with STARD principles requiring transparent reporting of the index test, reference standard, analysis, and indeterminate or missing results (Cohen et al., 2016).

Pathogen limit of detection should not be substituted for mutation limit of detection. Mutation-level sensitivity should not be substituted for drug-level sensitivity. Isolate performance should not be presented as direct-specimen performance. These distinctions are particularly important when a single targeted variant is common but does not explain all resistant phenotypes.

### 5.2 Standardized result categories

A useful laboratory report includes assay validity, the exact gene and variant, the drug-level evidence tier, the validated panel boundary, any low-frequency or mixed-allele signal, and the recommended confirmation pathway. As an author-proposed, non-consensus reporting model, we suggest four conceptual categories: valid positive, valid negative within coverage, low-frequency or mixed signal, and invalid or insufficient input. These categories are intended to make claim boundaries explicit and to stimulate future standardization; they are not current consensus laboratory standards or regulatory criteria. A categorical resistant call should be tied to the relevant WHO evidence tier, whereas a targeted negative applies only to the validated panel boundary. The corresponding data elements are listed in Supplementary Table S3.

For a valid positive result, the report should state that *Mycobacterium tuberculosis* nucleic acid and the specified validated resistance-associated variant were detected, identify the drug-level interpretation that is supported, and recommend broader testing for drugs or mechanisms outside the panel. For a valid negative result, it should state that no targeted resistance-associated variant was detected in the listed genes or regions within validated coverage and explicitly state that phenotypic susceptibility has not been established.

For a low-frequency or mixed-allele result, the report should identify the variant and signal category, state whether the signal exceeds the analytical and reproducible-reporting thresholds, and recommend repeat testing and orthogonal confirmation unless an outcome-supported clinical-action threshold has been established. For an invalid or insufficient-input result, drug-resistance interpretation should be withheld, the failed control or input limitation should be identified, and repeat or alternative testing should be recommended.

These categories are most useful when incorporated directly into the laboratory report rather than added as an optional comment. Local wording can be concise, but the panel boundary, the limits of inference, and the required follow-up should remain explicit. Core recommended validation and reporting elements are summarized in Table 5; the framework was proposed by the authors and is not a validated consensus reporting guideline.

**TABLE 5:** Author-proposed, non-consensus core validation and reporting elements for CRISPR-enabled drug-resistant tuberculosis studies.

Domain	Core reporting requirement	Why it matters	Unsupported inference	Recommended interpretation
Intended use and claim level	Define rule-in detection, triage, multiplex screening, sequencing enrichment, and the highest claim assessed	Different intended uses require different evidence	Do not infer that a single platform establishes universal drug-resistant tuberculosis diagnosis	Restrict conclusions to the prespecified claim level
Study and specimen type	State constructed, simulated, isolate, direct-specimen, or pathway evidence; report center number and blinding	Specimen realism determines evidentiary strength	Do not infer that isolate performance equals direct-specimen performance	State the exact evidence category
Bacillary load, controls, and invalid results	Report specimen input, bacillary-load category, extraction and reaction controls, and invalid rate	Low input increases false-negative and invalid results	Do not interpret a control-failed negative as valid	Repeat testing or use an alternative method
Mutation performance and panel coverage	Report variant-specific discrimination, minimum mutant fraction, genes and variants covered, and regional gaps	Pathogen detection differs from mutation detection, and negative calls apply only within coverage	Do not equate pathogen and mutation limits or infer susceptibility from hotspot absence	Report mutation and panel-level performance separately
Multiplex and enrichment bias	Assess primer competition, allele dropout, wild-type depletion, enrichment uniformity, and cross-target interference	Bias can alter allele fractions and complete-panel sensitivity	Do not assume that singleplex performance predicts multiplex performance	Validate the assembled panel in relevant backgrounds
Workflow, contamination, and traceability	Describe every tube opening, carryover control, internal control, lot, instrument, and threshold version	False resistance calls can lead to inappropriate treatment changes	Do not assume that one-pot operation automatically means closed sample-to-result testing	Define the workflow and invalid-result rules explicitly
Reference standards and minority signals	Use sequencing for exact variants; match drug-level claims to mutation catalogues and technically valid phenotypic testing; prespecify analytical, reporting, and action thresholds	Different comparators answer different questions	Do not assume that one comparator validates all claims or that any minority signal mandates a regimen change	Match the reference to the claim and confirm unvalidated minority signals
Result reporting and decision impact	Report valid positive, valid negative within coverage, mixed/low-frequency, or invalid results; assess treatment change, referral, confirmation burden, and time to effective therapy	Analytical performance does not establish clinical utility	Do not infer susceptibility from a negative compact panel or clinical benefit from reaction time alone	Use bounded report language and evaluate the complete diagnostic pathway

Abbreviations: pDST, phenotypic drug susceptibility testing. This author-proposed framework is intended to support claim-specific evaluation and is not a validated consensus reporting guideline.

### 5.3 Positioning relative to existing technologies

Based on the current evidence base, CRISPR-based testing is best viewed as a complement to established methods rather than as a universal competitor. WHO-recommended rapid molecular platforms provide automation, standardized quality control, and substantially stronger near-patient clinical evidence, although target content is fixed (World Health Organization, 2025c; Pfurtscheller et al., 2025; Penn-Nicholson et al., 2022; Horne et al., 2025; Pillay et al., 2022; Cao et al., 2021). Line-probe assays provide established hotspot coverage but require multistep workflows and expert interpretation (Nathavitharana et al., 2017). tNGS provides broad, updateable multi-gene resistance profiling and is explicitly incorporated into current WHO diagnostic pathways, although it depends on sequencing infrastructure, quality control, and variant interpretation (World Health Organization, 2025c; Schwab et al., 2024; Colman et al., 2025). A culture-free sequencing study of 96 MTBC-positive sputum specimens reported interpretable tNGS resistance profiles in 96.6% of evaluable samples with an approximate analytical limit near 10 genome copies, illustrating the breadth of the contemporary sequencing comparator rather than establishing a head-to-head advantage over CRISPR (Iannucci et al., 2026). Whole-genome sequencing offers broader genomic coverage but generally has greater input and workflow requirements, while phenotypic drug susceptibility testing measures expressed resistance, including mechanisms outside known mutation panels, but remains culture dependent (Heyckendorf et al., 2018; Yusoof et al., 2022; Walker et al., 2015; Coll et al., 2015). A practical comparison is provided in Supplementary Table S4.

These ceilings should also be compared explicitly with the measured clinical sensitivity of assays already used in WHO-recommended pathways. A Cochrane review of Xpert MTB/XDR reported summary sensitivities of 94.2% (95% CI 87.5–97.4) for isoniazid resistance and 93.2% (88.1–96.2) for fluoroquinolone resistance against phenotypic drug susceptibility testing (Pillay et al., 2022). A 2024 systematic review and meta-analysis of tNGS reported pooled rifampicin-resistance sensitivity of 99.1% (95% credible interval 98.3–99.7) (Schwab et al., 2024). These estimates are not head-to-head with the region-level benchmarks and arise from different study populations and reference frameworks; nevertheless, their measured sensitivities are higher than the corresponding 85.4% isoniazid, 84.7%–88.2% fluoroquinolone, and 92.6% rifampicin comparator ceilings used here. Because a finite allele-specific CRISPR guide set can only match or fall below the relevant ceiling, the principal translational case for a compact CRISPR panel is not superior resistance coverage. It instead rests on complete sample-to-result turnaround time, total pathway cost, decentralization, rapid rule-in/triage, and, in a separate use case, sequencing enrichment.

The clearest potential advantages of CRISPR are programmability for a limited number of region-specific targets, rapid rule-in of selected highly actionable variants, potential minority-variant interrogation when molecular input is adequate, and targeted enrichment before sequencing. These are platform-dependent potential advantages rather than demonstrated superiority over Xpert or tNGS. Comparative studies should therefore measure complete sample-to-result turnaround time, total pathway cost, invalid rates, decentralization requirements, and decision impact rather than isolated reaction time. Cas9-guided enrichment occupies a distinct interface between rapid molecular testing and comprehensive sequencing (Tram et al., 2023).

### 5.4 Integration into the diagnostic and treatment pathway

Clinical placement should begin from the current WHO-aligned pathway rather than from CRISPR itself. For people with presumptive tuberculosis, WHO-recommended initial molecular testing can establish bacteriological confirmation and provide initial rifampicin-resistance information, followed by expanded drug-resistance testing and tNGS according to the result, patient risk, regimen requirements, and local availability (World Health Organization, 2025c). Within that established pathway, an author-proposed CRISPR module could serve only as an optional rapid rule-in/triage step for selected *rpoB*, *katG*/*inhA*-*fabG1*, or other validated variants. A positive WHO Group 1 result can trigger earlier entry into the appropriate resistance work-up, but the CRISPR result should feed back into established broader testing rather than directly define a complete regimen. An isoniazid-resistant, rifampicin-susceptible pathway should be assigned only after rifampicin susceptibility has been established by a validated WHO-recommended molecular or phenotypic method.

For confirmed or strongly suspected multidrug- or rifampicin-resistant tuberculosis, an optional rapid CRISPR fluoroquinolone rule-in module could target selected WHO-supported *gyrA*/*gyrB* variants. A positive validated variant can support fluoroquinolone-resistance inference and may contribute to pre-extensively drug-resistant tuberculosis classification after multidrug/rifampicin resistance is established; a negative compact panel should proceed to broader testing rather than be interpreted as susceptibility. Invalid/insufficient-input or low-frequency/mixed signals require repeat or orthogonal confirmation. A separate potential CRISPR niche is sequencing enrichment for low-MTBC-DNA specimens before tNGS/WGS. Figure 5 is an author-generated conceptual placement model: established WHO-aligned testing constitutes the clinical backbone, whereas CRISPR insertion points are proposals requiring prospective comparative validation.

**FIGURE 5:**
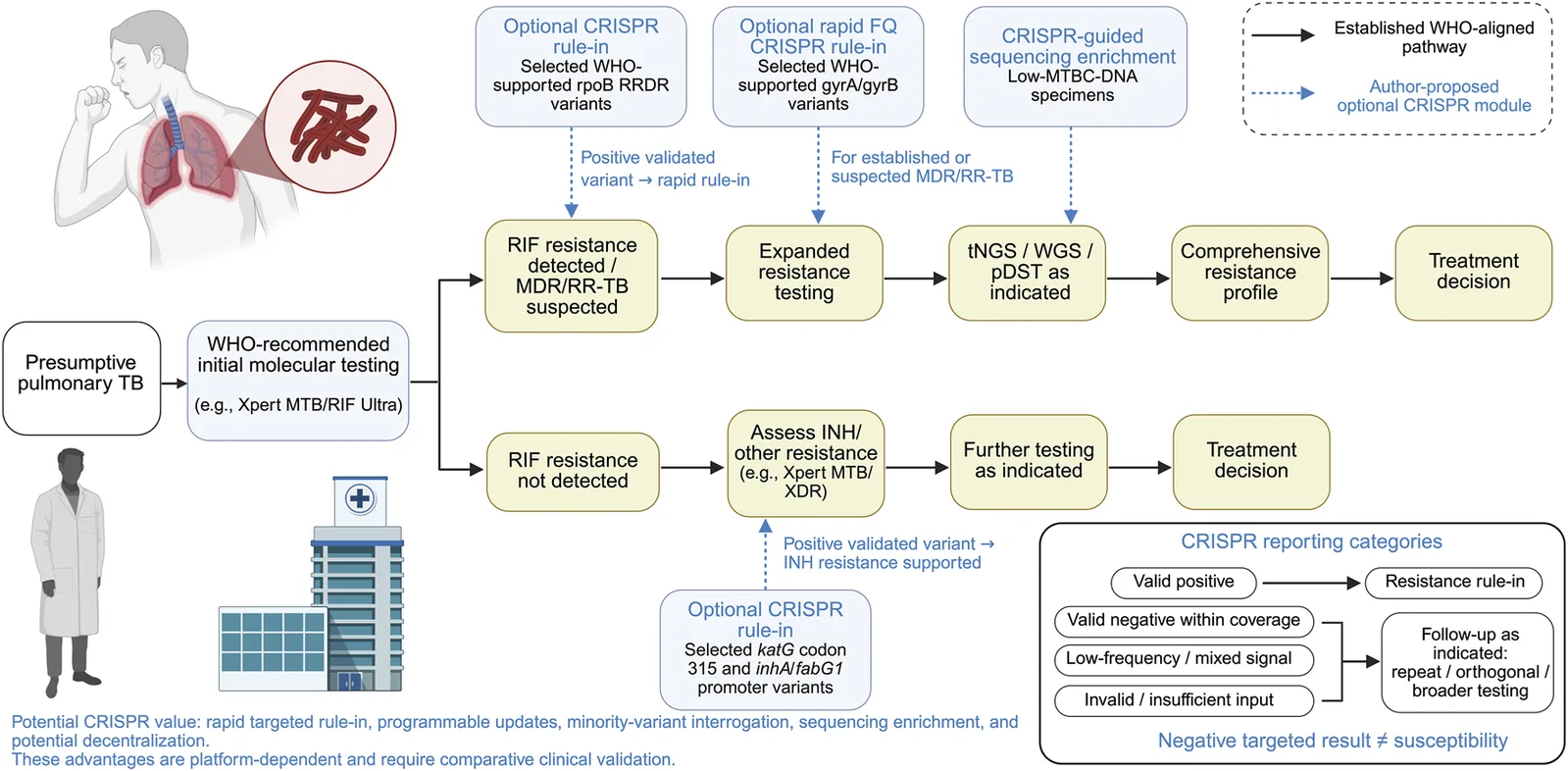
Author-generated conceptual staged clinical placement and reporting pathway for CRISPR-enabled drug-resistant tuberculosis testing. CRISPR is presented as a potential complement to WHO-recommended molecular testing and targeted next-generation sequencing, not as a WHO-endorsed replacement. Abbreviations: FQ, fluoroquinolone; INH, isoniazid; MDR/RR-TB, multidrug- or rifampicin-resistant tuberculosis; MTBC, *Mycobacterium tuberculosis* complex; pDST, phenotypic drug susceptibility testing; RIF, rifampicin; tNGS, targeted next-generation sequencing; WGS, whole-genome sequencing.

### 5.5 Decentralized implementation and quality assurance

Portability and potentially reduced equipment requirements do not establish point-of-care readiness. A decentralized CRISPR platform should be compared with complete operational workflows of existing near-patient options, including automated cartridge-based systems, line-probe assays, and increasingly portable or decentralized sequencing configurations, rather than with reaction chemistry alone. It should integrate specimen inactivation, lysis, amplification, detection, objective readout, and waste containment; include internal controls; and define training, invalid-result, retesting, referral, quality-assurance, and maintenance procedures. Candidate field formats include lateral flow, paper-based systems, portable fluorescence, and digital partitioning (Posthuma-Trumpie et al., 2009; Yin et al., 2021; Zhang et al., 2024; Wang et al., 2024). Supplementary Table S4 summarizes these implementation trade-offs.

Cost should be evaluated at the pathway level rather than as a single-reaction reagent estimate. Closed consumables, readers, quality-control materials, training, maintenance, repeat testing, waste disposal, and confirmation of positive or discordant results all contribute. False resistance calls can lead to the inappropriate exclusion of active drugs, while false-negative results can delay an effective regimen. Decision-impact and cost-effectiveness studies should therefore measure time to appropriate treatment, unnecessary drug exclusion, confirmation burden, and programmatic sustainability.

## 6 Discussion

### 6.1 Current clinical position

Current evidence spans variant-specific analytical discrimination, selected isolate-based validation, a small number of direct respiratory-specimen studies, one-tube multiplex rifampicin-variant screening, a small integrated sample-to-result *rpoB* genotyping evaluation, and CRISPR-assisted sequencing enrichment (Liu P. et al., 2025; Tram et al., 2023; Xiao et al., 2025; Wu G. et al., 2025; Wu et al., 2024; Wu Y. et al., 2025; Cheng et al., 2026; Hu et al., 2026; Bai et al., 2022; Gan et al., 2026). The focused peer-reviewed resistance-specific diagnostic literature remains small (13 studies in the evidence matrix used for this review), and no included study demonstrates prospective multicenter treatment-decision impact. The strongest current evidence supports candidate rapid rule-in detection of selected WHO-supported variants associated with rifampicin, isoniazid, and fluoroquinolone resistance. It does not support using compact negative panels to exclude resistance or replacing tNGS, WGS, and phenotypic drug susceptibility testing across the full resistance spectrum.

### 6.2 Key translational tensions

The principal translational tension is between rapid hotspot detection and incomplete rule-out coverage. Compact panels can provide clinically useful early evidence of resistance, but their apparent simplicity can encourage overinterpretation of negative results. A second tension lies between regional programmability and standardization. Updating guides may improve local coverage, yet each change can alter amplification balance, analytical thresholds, and report interpretation. An updatable panel therefore requires a defined revalidation process rather than simple replacement of individual guides.

A third tension concerns intended use. Stand-alone rapid assays and CRISPR-assisted sequencing enrichment solve different diagnostic problems and should not be ranked on a single point-of-care scale. The rolling-circle-amplification cassette and the integrated thin-film study show that one-tube multiplexing and sample-to-result automation are technically achievable, but their limited evaluations do not establish multicenter robustness, programmatic invalid rates, or treatment impact (Wu Y. et al., 2025; Cheng et al., 2026). FLASH-TB broadens sequencing access to low-abundance specimens but remains dependent on sequencing infrastructure and enrichment-quality control (Tram et al., 2023).

### 6.3 Controversies and unresolved questions

Several questions remain unresolved. First, no consensus clinical-action threshold exists for low-frequency resistant alleles, and any threshold is likely to depend on drug, regimen, specimen, total amplifiable input, and analytical method (Metcalfe et al., 2017; Dreyer et al., 2020; Colman et al., 2015; Folkvardsen et al., 2013; Operario et al., 2017; Ängeby et al., 2012). Second, sequencing and phenotypic drug susceptibility testing answer different questions, so no single comparator validates exact-variant detection, drug-level inference, and clinical utility simultaneously (Bossuyt et al., 2015; Miotto et al., 2017; Cohen et al., 2016; Coll et al., 2015). Third, regionally updatable panels may improve local coverage but complicate standardization, cross-site comparability, and version control (Walker et al., 2022; World Health Organizatio n, 2023). Prospective studies should therefore link prespecified thresholds and panel versions to treatment decisions and outcomes.

### 6.4 Limitations of the current evidence and this review

This narrative review integrates heterogeneous evidence from constructed targets, clinical isolates, direct specimens, and CRISPR-assisted sequencing studies. The search and study-selection approach is transparent but narrative rather than systematic, and no pooled diagnostic-accuracy estimate was calculated. Most resistance-specific CRISPR studies are small, geographically restricted, target-limited, or evaluated against sequencing without an independent phenotypic comparator; prospective multicenter reproducibility, invalid-rate, and treatment-outcome data are largely absent. Sequencing of the same amplified locus is appropriate for exact-sequence concordance but can share upstream amplification or allele-dropout errors with the index assay and does not independently validate drug-level sensitivity. Source-data inconsistencies were retained and explicitly identified rather than silently reconciled (Supplementary Table S5). For recent integrated studies, incomplete description of specimen composition or phenotype-level contingency data limits confidence-interval calculation and clinical generalization. The mutation-to-drug-to-decision framework itself is an author-proposed interpretive model, not a validated numerical score or consensus reporting system.

### 6.5 Priorities for engineering and clinical validation

Future engineering studies should prioritize preservation of mutant fractions during sample preparation, balanced multiplex amplification, reliable closed-tube workflows, objective readout, and internal controls that distinguish true negatives from insufficient input or inhibition. Clinical validation should include larger direct-specimen cohorts, bacillary-load stratification, multicenter testing, prospective designs, and treatment-linked outcomes. Regional panel updates should be based on mutation catalogues, local sequencing and phenotypic surveillance, and treatment-outcome evidence, followed by re-evaluation of multiplex interference, thresholds, reagent lots, and direct-specimen performance. Relevant endpoints include time to an effective regimen, changes in drug selection, confirmation burden, false resistance calls, inappropriate exclusion of active drugs, and total costs across the diagnostic pathway.

## 7 Conclusion

At present, the strongest current evidence supports candidate rapid rule-in assays for selected WHO-supported *rpoB*, *katG*, *inhA*/*fabG1*, and *gyrA*/*gyrB* resistance-associated variants, together with CRISPR-assisted enrichment for specimens with a low *Mycobacterium tuberculosis* DNA burden. Their plausible value lies in earlier resistance stratification, triage, and appropriately targeted confirmation, not in replacing WHO-recommended rapid molecular testing, tNGS/WGS, or phenotypic drug susceptibility testing. Interpretation remains bounded by specimen validity, absolute molecular input, exact panel coverage, mutant fraction, and the strength of variant evidence. Clinical translation will require transparent evidence grading, robust direct-specimen validation, balanced multiplex performance, closed workflows, standardized reporting, population-specific coverage analysis, quality assurance, and prospective demonstration of patient- or pathway-level benefit.
