# Supplementary material for: From mutation detection to treatment decisions: CRISPR-enabled diagnostics for drug-resistant tuberculosis
Source: Front Bioeng Biotechnol. 2026 Sep 17;14:1947704. doi: 10.3389/fbioe.2026.1947704 (PMC13626866; doi:10.3389/fbioe.2026.1947704)
Supplement: Supplementary file 1 [file DataSheet1.pdf]

Supplementary Material

Table S1. Evidence categories and the maximum diagnostic claim ordinarily supported by each category.

Category	Typical material or design	Primary purpose	Maximum claim ordinarily supported	Key reporting requirement
E1: Molecular engineering	Designed oligonucleotides, plasmids, purified targets	Guide selection, target accessibility, reporter optimization	Analytical feasibility for a defined target	Sequence context, concentrations, replicates, cross-reactivity
E2: Simulated allele mixtures	Wild-type and mutant templates mixed at known fractions	Minority-variant detection and wild-type interference	Analytical mutant-fraction threshold under controlled conditions	Expected and measured fractions, amplification bias, reproducibility
E3: Clinical isolates	DNA or RNA from cultured clinical isolates	Natural-genome variant concordance	Mutation-level performance in isolates	Lineage, target variants, sequencing comparator, phenotypic comparator
E4: Direct clinical specimens	Sputum, bronchoalveolar lavage, or other specimens tested without prior culture	Specimen processing and direct diagnostic accuracy	Direct-specimen targeted mutation performance	Bacillary load, input, internal controls, invalid rate, panel coverage
E5: Prospective pathway evidence	Multicenter or programmatic cohort linked to treatment decisions	Clinical utility and operational performance	Decision impact, time to treatment, referral, and cost	Prospective design, blinded comparators, treatment and outcome endpoints
X1: Transferable tuberculosis engineering	Direct-specimen CRISPR pathogen detection without resistance targets	Lysis, concentration, closed workflow, or readout feasibility	Engineering transferability only	Do not describe as drug-resistance validation
X2: Mutation discovery	CRISPR mutagenesis or computational guide design	Candidate resistance mechanism or panel design	Candidate-target generation	Independent genotype–phenotype and clinical validation required

Table S2. Population-level region-interrogation coverage benchmarks relevant to, but not equal to, the proposed compact CRISPR target architecture.

Drug / claim	Reference target region / architecture	Approximate phenotypic-resistance coverage benchmark	Residual resistance outside target set	Evidence basis / current CRISPR validation	Interpretation boundary
Rifampicin	*rpoB* RRDR (e.g., p.Ser450Leu, p.His445 substitutions, p.Asp435 substitutions); codons 426-452 modern (507-533 legacy)	92.6% (9,135/9,869; exact 95% CI 92.0-93.1)	At least ~7.4%	Coverage source/data: Brankin et al. 2022, WHO-catalogue-derived genotype-phenotype dataset (9,869 phenotypically RIF-resistant isolates); WHO 2023 catalogue. CRISPR evidence: small direct respiratory-specimen cohorts plus isolate-level rifampicin studies.	Region-interrogation comparator ceiling; not exact-guide panel coverage. A finite exact-variant guide set may cover less. Extra-RRDR Group 1 markers such as p.Val170Phe and p.Ile491Phe remain clinically relevant.
Isoniazid	*katG* p.Ser315Thr + clinically relevant *inhA*/*fabG1* promoter variants	85.4% directly documented for *katG* codon 315 + *inhA* promoter (1,003/1,174; exact 95% CI 83.3-87.4%); broader molecular target sets up to ~89.4%	~10.6-14.6% depending on exact target set	Coverage source/data: Dean et al. 2020 multicountry dataset (1,174 phenotypically INH-resistant isolates); Brankin et al. 2022; WHO 2023 catalogue. CRISPR evidence: isolate-level evidence plus limited direct respiratory-specimen evaluation.	The incremental contribution of *fabG1* cannot be isolated without mutation co-occurrence data; exact allele-specific panel coverage requires a finalized guide list.
Levofloxacin	*gyrA* QRDR (e.g., codons 90, 91, and 94) + selected drug-specific WHO Group 1/2 *gyrB* variants	84.7% (2,648/3,125; exact 95% CI 83.4-86.0)	~15.3%	Coverage source/data: Brankin et al. 2022, WHO-catalogue-derived genotype-phenotype dataset (3,125 phenotypically LEV-resistant isolates); WHO 2023 catalogue. CRISPR evidence: predominantly isolate-level targeted FQ evidence; established direct-sputum validation is lacking.	Upper-bound benchmark from wild-type-probe region interrogation (GenoType MTBDRsl v2: *gyrA* codons 85-96 + *gyrB* codons 497-502); this does not imply that allele-specific CRISPR interrogates the full regions. Exact guide selection, allele frequencies, and geography will alter panel coverage.
Moxifloxacin	*gyrA* QRDR (e.g., codons 90, 91, and 94) + selected drug-specific WHO Group 1/2 *gyrB* variants	88.2% (1,658/1,880; exact 95% CI 86.6-89.6)	~11.8%	Coverage source/data: Brankin et al. 2022, WHO-catalogue-derived genotype-phenotype dataset (1,880 phenotypically MXF-resistant isolates); WHO 2023 catalogue. CRISPR evidence: predominantly isolate-level targeted FQ evidence; established direct-sputum validation is lacking.	Upper-bound benchmark from wild-type-probe region interrogation (GenoType MTBDRsl v2: *gyrA* codons 85-96 + *gyrB* codons 497-502); this does not imply allele-specific CRISPR region-level interrogation. *gyrB* evidence is drug specific and some variants are associated with low-level resistance.

Panel-level coverage statement: an exact percentage is not estimable for the proposed CRISPR architecture because it is a conceptual target architecture rather than a finalized allele-specific guide panel. A panel-level estimate should be calculated only after the exact guide list is fixed and evaluated against a prespecified genotype-phenotype dataset.

Selected WHO-supported *gyrB* variants are drug specific and should be version controlled. Group 1 variants provide the strongest catalogue-supported rule-in evidence; Group 2 variants should be identified explicitly as interim evidence, while Group 3 variants are of uncertain significance and should not support an unqualified resistant call.

Key sources for Table S2:

Brankin A, Seifert M, Georghiou SB, Walker TM, Uplekar S, Suresh A, et al. In silico evaluation of WHO-endorsed molecular methods to detect drug resistant tuberculosis. Scientific Reports. 2022;12:17741. doi:10.1038/s41598-022-21025-6.

Dean AS, Zignol M, Cabibbe AM, Falzon D, Glaziou P, Cirillo DM, et al. Prevalence and genetic profiles of isoniazid resistance in tuberculosis patients: a multicountry analysis of cross-sectional data. PLoS Medicine. 2020;17(1):e1003008. doi:10.1371/journal.pmed.1003008.

World Health Organization. Catalogue of mutations in *Mycobacterium tuberculosis* complex and their association with drug resistance. 2nd ed. Geneva: World Health Organization; 2023.

Chen Y, et al. Advantages of updated WHO mutation catalog combined with existing whole-genome sequencing-based approaches for *Mycobacterium tuberculosis* resistance prediction. Genome Medicine. 2025;17:31.

Table S3. Detailed data elements for an author-proposed, non-consensus standardized CRISPR drug-resistance report.

Element	Required content	Example
Specimen and assay validity	Specimen type, input or bacillary-load category, internal-control result, valid/invalid status	Sputum; low bacterial load; extraction and reaction controls passed; valid
Pathogen signal	Whether *Mycobacterium tuberculosis* input was detected above the validated threshold	*Mycobacterium tuberculosis* detected
Exact mutation call	Gene, nucleotide or amino-acid change, numbering convention, and signal category	*rpoB* p.Ser450Leu (legacy S531L), categorical positive
Drug-level evidence	Drug, WHO catalogue group, and whether the result supports categorical rule-in or interim/uncertain interpretation	WHO Group 1: associated with resistance; rifampicin resistance rule-in supported. Group 2: interim evidence stated explicitly. Group 3: uncertain significance; no unqualified resistant call.
Panel boundary	Genes, regions, and variants included in the assay	Validated targets include selected *rpoB* resistance-determining region variants only
Minority signal	Estimated fraction or signal ratio, total amplifiable MTBC input, analytical threshold, reporting threshold, and any independently validated action threshold	Mixed-allele signal above analytical threshold with adequate input; clinical-action threshold not established
Negative-language boundary	Explicit statement that absence of targeted variants does not establish susceptibility	No targeted variant detected within coverage; phenotypic susceptibility not established
Recommended action	Repeat testing, expanded panel, sequencing, phenotypic testing, or treatment review	Proceed to targeted sequencing and, where technically validated and clinically interpretable, phenotypic drug susceptibility testing
Traceability	Reagent lot, instrument, operator or run identifier, and software or threshold version	Panel version 2.1; lot 24A; threshold set v3

Table S4. Practical positioning of CRISPR-enabled testing relative to established drug-resistant tuberculosis technologies.

Technology	Main strength	Principal limitation	Most appropriate claim	Potential role of CRISPR	Confirmation or dependency
Xpert MTB/RIF or Ultra	Automated near-patient tuberculosis and rifampicin-resistance testing with strong clinical evidence	Fixed target content and limited broader resistance coverage	Rapid tuberculosis and rifampicin-resistance detection	Add flexible region-specific or secondary resistance rule-in targets	Follow current diagnostic pathway for expanded resistance testing
Xpert MTB/XDR	Automated detection of selected isoniazid, fluoroquinolone, ethionamide, and injectable-drug loci	Fixed panel and incomplete coverage of all resistant phenotypes	Rapid targeted expanded resistance detection	Complement with locally updatable targets or pre-sequencing triage	Sequencing or phenotypic testing for uncovered mechanisms
Line-probe assay	Established hotspot detection across several drugs	Multistep workflow and interpretive expertise	Targeted mutation-based resistance detection	Potential simplified or closed-tube alternative for selected variants	Phenotypic or sequence confirmation as indicated
Targeted next-generation sequencing	Broad multi-gene mutation profile, updateable interpretation, and WHO-aligned role in follow-on DST	Sequencing infrastructure, cost, quality control, and variant-interpretation requirements	Broad genotype-based resistance prediction; a 2026 culture-free study obtained interpretable profiles in 96.6% of evaluable sputum specimens with an approximate ~10-genome-copy LoD (157)	CRISPR may provide optional rapid triage before sequencing or targeted enrichment of low-abundance specimens	WHO mutation catalogue and validated interpretation; phenotypic testing for selected uncertain/discordant situations
Whole-genome sequencing	Comprehensive genomic coverage and surveillance value	Greater input and workflow requirements, particularly for direct specimens; complex interpretation	Comprehensive known-variant profiling and epidemiology	Targeted enrichment of low-abundance direct specimens	Culture or targeted enrichment may be required; correlate uncertain calls with phenotypic evidence where technically valid
Phenotypic drug susceptibility testing	Measures the expressed resistance phenotype, including some mechanisms outside known mutation panels	Slow, culture-dependent, technically demanding, and not equally mature or interpretable for every drug	Drug-level susceptibility or resistance phenotype	Prioritize samples and provide early rule-in before phenotype result	Important for discordant, rare, or newer-drug resistance where technically validated and clinically interpretable
Rapid compact CRISPR panel	Programmable, potentially decentralized, variant-specific rule-in	Limited panel coverage and risk of overinterpreting negatives	Rapid detection of selected, well-validated resistance-associated variants	Primary CRISPR use case	Broader molecular or phenotypic confirmation

Technology	Main strength	Principal limitation	Most appropriate claim	Potential role of CRISPR	Confirmation or dependency
CRISPR-assisted sequencing enrichment	Improves target read recovery in low-abundance specimens	Requires sequencing and enrichment-quality control	Improved sequencing success and depth	Bridge between rapid assays and comprehensive sequencing	Sequencing infrastructure and validated variant interpretation

Table S5. Expanded comparative summary of representative CRISPR-enabled studies relevant to drug-resistant tuberculosis.

Approach	Cas role	Targets	Evidence material	Reported scale or metric	Claim supported	Important boundary
Engineered Cas12a RR for *rpsL* (65)	Expanded-PAM Cas12a collateral readout	*rpsL* p.Lys43Arg/p.Lys88Arg	Constructed/simulated mixtures	Mutant fraction reported down to approximately 0.1%	Analytical low-frequency discrimination	No direct-specimen or clinical-action threshold; total amplifiable input is required to interpret the reported mutant fraction
Engineered Cas12a RR + WT depletion (66)	Variant detection after restriction-enzyme depletion	*katG* p.Ser315Thr	Constructed mixtures and 42 cultured isolates (27 mutation-positive; 15 negative)	~1% minority fraction; isolate exact-variant Se 100% (27/27; 95% CI 87.2-100) and Sp 100% (15/15; 78.2-100)	Analytical enrichment + isolate exact-variant discrimination	No direct-specimen validation; one INH variant; WT depletion alters allele fractions; full drug-level sensitivity not established; total amplifiable input is required to interpret the reported mutant fraction
Asymmetric RPA-Cas12a (67)	Single-stranded target generation	Selected RIF variants	Simulated mixtures	~1% mutant fraction; LoD 1 fM; 2 µL input ≈1.2×10^3 target molecules/reaction.	PAM-independent analytical discrimination	Clinical-specimen inhibition and amplification bias untested
Multiplex RPA-AaCas12b (118)	Collateral readout after multiplex RPA	IS6110, *rpoB* c.1349C>T, *katG* c.944G>C	48 residual, pretreated sputum/BALF specimens	Sequencing: *rpoB* Se 94.1% (16/17; 71.3-99.9), Sp 100% (31/31; 88.8-100); *katG* Se 94.7% (18/19; 74.0-99.9), Sp 93.1% (27/29; 77.2-99.2). Phenotype sensitivity lower.	Direct-specimen exact-mutation detection	~40-min preparation precedes 30-35-min assay; limited allele specificity toward resistance-associated *katG* p.Ser315Asn; one variant per drug; readout threshold affects classification
Cas13a FQ assay (127)	RNA collateral readout after PCR/T7 transcription	Seven common *gyrA* variants	75 cultured clinical isolates	Se 91.4% (32/35; 95% CI 76.9-98.2) and Sp 100% (40/40; 91.2-100) versus pDST	Targeted FQ-resistance rule-in in isolates	No direct sputum; incomplete *gyrB* and mechanism coverage
Multiplex PCR-Cas14a - Xiao (77)	PAM-independent ssDNA recognition	Selected *rpoB*, *katG*, *inhA* hotspots	60 isolates; 55 smear-positive, culture-positive sputum specimens	Isolate RIF: 39/42 = 92.9%, whereas the source reports 93.3% (95% CI 80.0-98.5). Sputum RIF Se 83.3% (10/12; 51.6-97.9), Sp 100% (43/43; 91.8-100); INH Se 100% (11/11; 71.5-100), Sp 100% (44/44; 92.0-100). Source reports 85.7% for the same 10/12 sputum RIF count.	Direct-sputum targeted hotspot detection	High-bacillary-load cohort; open transfer; two source arithmetic inconsistencies retained (39/42 reported as 93.3% and 10/12 reported as 85.7%); *rpoB* p.Asp435Tyr miss despite nominal guide inclusion (possible analytical/source inconsistency); p.Leu452Pro not covered
Asymmetric PCR-Cas14a - Wu (78)	Eight mutation-specific Cas14a guides	Eight prevalent *rpoB* RRDR mutation types	16 cultured clinical isolates	128 blinded reactions; concordant with sequencing	Multigenotype *rpoB* detection in isolates	Extracted isolate DNA; no direct-specimen validation or clinical comparison with standard tests
CARP (75)	Cas9 cleavage followed by qPCR	*rpoB* p.Ser450Trp (legacy S531W) / p.His445Cys (legacy H526C)	Designed WT/mutant amplicons; no clinical cohort	Analytical discrimination; ~2 h cleavage + ~2 h qPCR. Source 0.0001 fg genomic DNA ≈2×10^-5 H37Rv genome equivalents, below plausible molecular-sampling limits.	Analytical variant discrimination	Two-variant coverage; no direct-specimen or clinical validation
ACURAT (76)	Nested PCR + PAM-PCR + micro-mismatch Cas9	15 *rpoB* genotypes at modern codons 435/445/450/452 (legacy 516/526/531/533)	60 culture-derived clinical isolates (10 drug-sensitive; 50 RIF/INH-resistant); CRISPR cross-validation on selected WT + six mutant isolates	Local 50-isolate RRDR survey; 94% of resistant-sample mutations occurred at modern codons 450/445/435 (legacy 531/526/516); no full-cohort ACURAT accuracy estimate	Expanded *rpoB* genotyping	Culture-derived/local-genotype evidence, not direct-specimen accuracy; complex multistep workflow

Approach	Cas role	Targets	Evidence material	Reported scale or metric	Claim supported	Important boundary
Multi-AS-PCR-Cas12a (84)	Allele-specific amplification + collateral readout	Ten *rpoB* variants	24 sputum/BALF specimens (9 RIF-resistant; 15 RIF-sensitive)	Se 100% (9/9; 66.4-100), Sp 93.3% (14/15; 68.1-99.8) vs sequencing; LoD 10^{2} aM (~10^{2} copies/μL); 5 μL input ≈3×10^{2} molecules/reaction.	Expanded targeted RIF hotspot detection	Ten primers could not function as one balanced mixture; C4/C6 grouping required; one false positive; small cohort
FLASH-TB (74)	Cas9-guided sequencing enrichment	Identification regions and 52 candidate resistance genes	16 direct sputum specimens	Resistance prediction in 15/16; increased depth	Improved direct-specimen sequencing interpretability	Sequencing dependence; small cohort; enrichment uniformity and allele fidelity
iCasdrop (80)	Dual Cas12a/Cas13a ratio calibration	Generic single-nucleotide-variant model	Methodological/simulated samples	Ratio correction of wild-type background	Transferable quantitative calibration	Not tuberculosis-specific clinical evidence
Cas9 electrochem. katG sensor (37)	Guide-directed cleavage converted to current change	*katG* codon 315	Designed DNA targets	Analytical limit 7.55 aM; copies/reaction not derivable from the reported reaction input.	Analytical mutation sensing	No direct clinical validation
RPA-Cas12a *rpoB* p.Leu378Arg (110)	Variant-specific collateral readout	*rpoB* p.Leu378Arg (WHO Group 3; uncertain significance)	Constructed or reference genomic materials; no direct clinical-specimen cohort	Variant-specific analytical performance	Panel updatability for a noncanonical rifampicin-resistance variant	WHO Group 3 uncertain significance (105); technical programmability only; no direct clinical-specimen validation or drug-phenotype sensitivity estimate
RCA-Cas12a-Multi-SNV cassette (87)	Padlock-probe ligation, rolling-circle amplification, Cas12a collateral readout	Four *rpoB* resistance-associated SNVs	16 sputum/BALF specimens	Source Se 100%, Sp 83.3%, PPV 90.9%, NPV 100%; implied Se 10/10 (69.2-100), Sp 5/6 (35.9-99.6); source table internally inconsistent; LoD 10^{4} aM (10 fM) in a 20-μL reaction ≈1.2×10^{5} target copies/reaction.	One-tube targeted RIF-variant screening	Four-variant coverage; primary extraction not integrated; source-data inconsistency limits independent reconstruction of clinical counts
Thin-film microfluidic CRISPR (88)	Alcohol-free extraction; multiplex AS-PCR; PAM-independent Cas12a dual-signal readout	Seven *rpoB* RRDR mutations	28 clinical specimens; composition not clearly reported	Full concordance with Sanger reported; class-specific 95% CI not derivable. 1 CFU/mL on-chip; 0.1 copies/μL at 20% mutant fraction; 20 μL extract ≈2 total copies (~0.4 mutant copy), highlighting sampling dependence.	Integrated targeted RIF genotyping	78-min sealed workflow; restricted panel; unclear clinical specimen composition; phenotype-level coverage, invalid rates, and multicenter reproducibility unestablished
CAMPER (146)	CRISPR-guided mutagenic polymerase	Candidate resistance loci including *atpE*	Experimental mycobacterial model	Functional support for the existing *atpE* p.Ile66Met resistance association (*M. tuberculosis* numbering)	Mutation discovery	Not diagnostic accuracy evidence
In silico regional guide design (147)	Computational guide selection	Candidate *gyrA*, *rpoB*, *katG*, *rpsL*, *embB*, and *ethA* targets	Genome databases	Predicted designability and specificity	Panel-design hypothesis	Laboratory, genotype–phenotype, and direct-specimen validation remain required
Cas9n/Cas9 self-priming assay - Gan (151)	Dual Cas9n/Cas9 recognition + PS-THSP signal amplification	*rpoB* RRDR	20 cultured clinical isolates (6 RIF-resistant; 14 susceptible)	100% concordance with pDST/PCR; Se 6/6 (54.1-100), Sp 14/14 (76.8-100); LoD 0.74 fM in 20 μL ≈8.9×10^{3} target molecules/reaction.	Isolate-level RIF-resistance screening	No direct specimens; resistant mutation spectrum not enumerated; self-priming amplification 100 min plus additional procedural steps
One-pot biplex RPA-Cas12a tongue-swab assay (152)	One-pot Cas12a pathogen detection	IS1081 and IS6110 (no resistance target)	Clinical tongue swabs; pathogen-detection study	Analytical sensitivity 1.1 genomes per 15-μL reaction; ~30-min sample-to-result	Transferable specimen/workflow engineering only	Does not interrogate resistance variants; should not be counted as DR-TB mutation validation

Abbreviations: BALF, bronchoalveolar lavage fluid; FQ, fluoroquinolone; gRNA, guide RNA; INH, isoniazid; PAM, protospacer-adjacent motif; PCR, polymerase chain reaction; pDST, phenotypic drug susceptibility testing; RIF, rifampicin; RPA, recombinase polymerase amplification; RRDR, rifampicin resistance-determining region; SNV, single-nucleotide variant; WT, wild type. Exact 95% CIs were calculated with the Clopper-Pearson method when explicit numerators and denominators were available. Sequencing-based metrics indicate exact-variant concordance, not necessarily drug-level sensitivity. Preprints were excluded. Cas14a nomenclature is retained where used in the source publication; these systems are now generally classified within Cas12f. Source concentration units are retained; approximate molecules/reaction are added only when input volume is unambiguous. Values below plausible molecular sampling are explicitly flagged.
